# Fractal and Multifractal Properties of Electrographic Recordings of Human Brain Activity: Toward Its Use as a Signal Feature for Machine Learning in Clinical Applications

**DOI:** 10.3389/fphys.2018.01767

**Published:** 2018-12-10

**Authors:** Lucas G. Souza França, José G. Vivas Miranda, Marco Leite, Niraj K. Sharma, Matthew C. Walker, Louis Lemieux, Yujiang Wang

**Affiliations:** ^1^Department of Clinical and Experimental Epilepsy, UCL Queen Square Institute of Neurology, University College London, London, United Kingdom; ^2^Institute of Physics, Federal University of Bahia, Salvador, Brazil; ^3^Interdisciplinary Computing and Complex BioSystems (ICOS) Research Group, School of Computing, Newcastle University, Newcastle upon Tyne, United Kingdom; ^4^Institute of Neuroscience, Newcastle University, Newcastle upon Tyne, United Kingdom

**Keywords:** EEG, fractal, multifractal, epilepsy, variance, characteristic time, signal features, machine learning

## Abstract

The quantification of brain dynamics is essential to its understanding. However, the brain is a system operating on multiple time scales, and characterization of dynamics across time scales remains a challenge. One framework to study such dynamics is that of fractal geometry; and currently there exist several methods for the study of brain dynamics using fractal geometry. We aim to highlight some of the practical challenges of applying fractal geometry to brain dynamics—and as a putative feature for machine learning applications, and propose solutions to enable its wider use in neuroscience. Using intracranially recorded electroencephalogram (EEG) and simulated data, we compared monofractal and multifractal methods with regards to their sensitivity to signal variance. We found that both monofractal and multifractal properties correlate closely with signal variance, thus not being a useful feature of the signal. However, after applying an epoch-wise standardization procedure to the signal, we found that multifractal measures could offer non-redundant information compared to signal variance, power (in different frequency bands) and other established EEG signal measures. We also compared different multifractal estimation methods to each other in terms of reliability, and we found that the Chhabra-Jensen algorithm performed best. Finally, we investigated the impact of sampling frequency and epoch length on the estimation of multifractal properties. Using epileptic seizures as an example event in the EEG, we show that there may be an optimal time scale (i.e., combination of sampling frequency and epoch length) for detecting temporal changes in multifractal properties around seizures. The practical issues we highlighted and our suggested solutions should help in developing robust methods for the application of fractal geometry in EEG signals. Our analyses and observations also aid the theoretical understanding of the multifractal properties of the brain and might provide grounds for new discoveries in the study of brain signals. These could be crucial for the understanding of neurological function and for the developments of new treatments.

## 1. Introduction

Brain dynamics are non-linear and are often considered as one of the most complex natural phenomena, involving several different and interacting temporal scales. For example, fast electric activity, slower chemical reactions, and even slower diffusive processes have been observed in the brain. Interestingly, brain dynamics have also been characterized as “scale-free” (Stam and de Bruin, 2004; Fraiman and Chialvo, 2012), meaning that certain signal properties stay preserved across different time scales. To describe and quantify such time scale invariant dynamics, the framework of fractal geometry is often applied (Werner, 2010).

Fractals have two specific properties: they consist of parts that are similar to the whole—termed self-similarity, and they have a fractional Hausdorff-Besicovitch dimension, also called fractal dimension (FD) (Mandelbrot, 1982; Feder, 1988; Falconer, 2003). Fractal geometry has been applied to the study of temporal dynamics, such as human brain dynamics in health (Lutzenberger et al., 1995; Pereda et al., 1998; Eke et al., 2000, 2002; Linkenkaer-Hansen et al., 2001; Bullmore et al., 2003, 2009; Gong et al., 2003; Acharya et al., 2005; Bassett et al., 2006, 2010; Hsu et al., 2007; Van De Ville et al., 2010; Papo et al., 2017) and disease (Esteller et al., 1999; Gómez et al., 2009; Zappasodi et al., 2014), providing intriguing results. For example, FD has been shown to vary prior to and during epileptic seizures (Esteller et al., 1999).

Objects adequately characterized by a single fractal dimension are referred to as monofractals. However, the fractal formalism has to be extended to capture certain phenomena that cannot be described by a single fractal dimension; these are called multifractals (Stanley et al., 1999). Multifractal objects can be conceived as decomposable into different subsets or parts, each characterized by its own distinct fractal dimension. The subsets are more precisely described as different statistical moments, and a multifractal is an object where the fractal dimension depends on the statistical moment being examined (Mukli et al., 2015). Thus multifractal objects are often described by a spectrum, showing the subsets/statistical moments and their corresponding fractal dimensions. Some natural phenomena exhibit multifractal patterns in space, for example, turbulence (Meneveau and Sreenivasan, 1987; Chhabra and Jensen, 1989), soil composition (Miranda et al., 2006; Zeleke and Si, 2006; Vázquez et al., 2008; Paz-Ferreiro et al., 2010a,b); and in time, for example heart beat patterns (Ivanov et al., 1999), and human physical activity (França et al., 2019).

There is also considerable evidence that brain dynamics are multifractal (Suckling et al., 2008; Ihlen and Vereijken, 2010; Ciuciu, 2012; Zorick and Mandelkern, 2013; Zhang et al., 2015; Papo et al., 2017; Xue and Bogdan, 2017; Racz et al., 2018). At the very least, additional statistical moments appear to be required, to characterize such dynamics (Fraiman and Chialvo, 2012). Furthermore, it is known that interacting processes with different time scales, similar to those observed in the brain, can generate multifractal patterns (Argoul et al., 1989; Suckling et al., 2008).

To measure the multifractal spectrum in brain dynamics, Multifractal Detrended Fluctuation Analysis (MF-DFA) (Kantelhardt et al., 2002) is currently the most used approach (Ihlen, 2012; Zhang et al., 2015). However, more advanced and potentially more stable estimation techniques have been proposed, such as the Multifractal Detrended Moving Average (Xu et al., 2017), and Chhabra-Jensen approaches (Chhabra and Jensen, 1989). These techniques, to our knowledge, however, have not yet been evaluated with brain signals. In addition, there are several parameter choices to be made for the purpose of the analysis. For example, to capture time-varying changes in multifractal properties, the epoch length and sampling frequency have to be chosen. These parameters may impact the multifractal estimation (Eke et al., 2002), but, to date, have not been studied systematically in the context of brain dynamics.

The biggest gap in the literature so far, however, is how multifractal properties relate to existing time series signal measures of brain dynamics (e.g., variance of the signal, band power, etc.). A major concern is that complex methods of analysis may not offer a significant advance over simpler, already established methods—this is crucial to a putative feature in machine learning applications, e.g., seizure prediction or detection (Mormann et al., 2007; Freestone et al., 2015; Brinkmann et al., 2016; Baldassano et al., 2017; Karoly et al., 2017; Kuhlmann et al., 2018a,b; Varatharajah et al., 2018). For example, in the analysis of the electroencephalogram of epileptic seizures, complex methods were found to actually reproduce patterns detected by simpler measures such as variance of the signal (Martinerie et al., 2003; McSharry et al., 2003). It is therefore, essential to understand how the (mono- and multi-) fractal measures relate to more traditional measures, and if new features can be obtained from the signal by applying a mono- or multi-fractal formalism.

To summarize, there is a knowledge gap in three critical areas: (1) which (multi)fractal characterization methodology is best suited for brain signals? (2) what are the optimal estimation parameters (e.g., in terms of recording epoch length) of potentially time varying multifractal properties? (3) what is the relationship between (multi)fractal properties and more traditional and established time series signal measures? To address these questions, we chose to analyse intracranially recorded human electroencephalography (icEEG) data, due to its high temporal resolution and high signal to noise ratio.

## 2. Materials and Methods

To address the questions above, we will first outline four experiments. We will then provide details on monofractal and and multifractal estimation methods, and also show how time series data with known mono- and multifractal properties can be generated to test the performance of the estimation methods. To test the multifractal measures on real-life brain signals, we then applied our analysis on human intracranial EEG. Thus, finally, we will summarize the EEG datasets used in this work.

The original scripts used in this work are available in https://github.com/yujiangwang/MultiFractalEEG. In addition, the following software packages were used: MATLAB; R (R Core Team, 2017); and ggplot2, R.matlab, reshape2, PerformanceAnalytics, and RColorBrewer (Wickham, 2007, 2009; Neuwirth, 2014; Peterson and Carl, 2014; Bengtsson, 2016).

### 2.1. Experiments

#### 2.1.1. Experiment 1: Monofractal Estimation With Respect to Changing Signal Variance

Estimation of monofractal properties has been applied to EEG signals in the past with varying and often contrasting results (Esteller et al., 1999; Li et al., 2005). A particular concern is that complex measures may simply reflect simple properties of the signal (Martinerie et al., 2003; McSharry et al., 2003). Hence, in our first analysis, we focus on the relationship between monofractal measures and signal variance. For this, we used a simulated monofractal time series (termed fractional Brownian motion, or short fBm) with its standard deviation modulated by a modified ramp function.

The fBm was simulated with a Hurst exponent *H* = 0.7 and a modulating function M (described in more detail later and in Appendix A in Supplementary Material) and split into 1,800 1,024-sample epochs. We estimated the monofractal dimension of this simulated signal using the Higuchi and DFA methods. To assess the impact of signal variance, we have also tested the effect of epoch-based standardization. To ensure that our effects were not simply an artifact generated by the fBm, we also repeated the analysis on one exemplary icEEG data segment.

#### 2.1.2. Experiment 2: Multifractal Estimation Stability

In order to evaluate the stability of multifractal properties in time, we generated a time series exhibiting stable multifractal properties over time using the p-Model. The time series was then evaluated using an epoch-based approach with the three estimators: MF-DFA, MF-DMA, and Chhabra-Jensen. The stability of the estimator can then simply be assessed as the temporal variability of its output.

#### 2.1.3. Experiment 3: Multifractal Estimation of Human EEG and Its Potential Added Value

To assess whether the chosen multifractal metrics contribute any non-redundant features about the signal in addition to more established signal metrics, we analyzed human EEG signals recorded intracranially. Again, we used an epoch-based approach, and we compared the multifractal metrics to a number of conventional signal metrics (mean, standard deviation, line length, bandpower) on each epoch. The similarity between signal features was evaluated using Pearson correlation and Mutual Information (Guyon and Elisseeff, 2003) (the code is available at https://github.com/robince/gcmi) (Ince et al., 2017). Furthermore, monofractal metrics were also included in this comparison, to further demonstrate the advantages in applying a multifractal over monofractal approaches.

#### 2.1.4. Experiment 4: Impact of Sampling Frequency and Epoch Length on Multifractal Estimation of Human EEG

Finally, we also evaluated the impact of the multifractal estimation parameters in the characterization of a seizure. We used intracranial EEG signals recorded from four patients undergoing pre-surgical planning, the signals were originally sampled at 5,000 Hz. For this analysis, down-sampled versions were evaluated with epochs of different sizes. To assess the effect of sampling frequency, we down-sampled the signal to 4,000, 3,000, 2,500, 2,000, 1,000, 800, 750, 600, 500, 400, 300, and 250 Hz. For each sampling frequency, we evaluated different epoch sizes (1,024, 2,048, 4,096, 8,192, and 16,384 points).

We defined a difference in multifractal spectrum width (Δα^†^) during the seizure compared to the background as the effect size (Cohen's D) between the ictal and interictal periods:

(1) $$\begin{array}{c} D=\frac{<\Delta \alpha_{ictal}^{†}>\;-\;<\Delta \alpha_{interictal}^{†}>}{s(\Delta \alpha_{interictal}^{†})} \end{array}$$

where < Δα^†^ > represents the mean and *s* denotes standard deviation.

### 2.2. Fractal Dimension Estimation

To estimate the monofractal properties from a time series, we used two established estimation approaches: Higuchi method (Higuchi, 1988) and Detrended Fluctuation Analysis (Peng et al., 1994). These methods are widely applied in the literature and aim to capture the features of a time series in a single scaling exponent.

Mandelbrot (1982) defined fractals as self-similar structures with fractal dimensions (*FD*) that are between their topological and embedding dimensions *T* and *E*, and an established relationship of *FD*+*H* = *E*, where *E* = *T*+1, *T* = 1 in the case of a time series, and *H* is the Hurst exponent. When assuming this self-similarity, we can measure both *FD* and *H* in our EEG time series as alternative ways of estimating the fractal dimension.

However, we also note that more generally, the fractal dimension *FD* and the Hurst exponent *H* do not necessarily reflect the same property of the time series (see Gneiting and Schlather, 2004 for more details). Indeed, we empirically tested the relationship between our estimated *FD* and *H* for an example EEG time series and found that *FD* and *H* correlate with ρ = −0.8, and their empirical relationship is *FD* = −0.86*H*+2.74. For our application in EEG time series, we conclude that *FD* and *H* measure two related, but slightly different signal properties [*FD*: a measure of roughness, *H*: a measure of long memory dependency (Gneiting and Schlather, 2004)]. Note that this of course also depends on how the *FD* and *H* are estimated exactly. Nevertheless, for our paper, we will apply these two established methods to estimate *FD* and *H*, respectively. We will assess the properties of both methods in the context of EEG, to demonstrate that our conclusions generalize to both types of measures.

#### 2.2.1. Higuchi Method

The Higuchi method measures the fractal dimension FD of a time series. It consists of constructing series with elements of an original time series and measuring their lengths (Higuchi, 1988). Given a time series with *N* time points *X*(1), *X*(2), …, *X*(*N*), the Equation (2) shows a rule for reconstructing smaller time series with elements of the original recording. The lengths of the time series can be assessed according to Equation (3). The brackets ⌊⌋ represent Gauss' notation, i.e., the rounded integer of the division (Higuchi, 1988). The variable *d* represents a down-sampling factor of the original time series.

(2) $$\begin{array}{c} \begin{array}{l} X(m),X(m+d),X(m+2d),\ldots ,X\left(m+\left[\frac{N-m}{d}\right]d\right)\text{ where} \end{array}\\ \;m=1,2,\ldots ,d \end{array}$$

(3) $$L_{m}(d)=\frac{\left\{\sum_{i=1}^{\left[(N-m)/d\right]}\left|X(m+id)-X(m+(i-1)d)\right|\frac{N-1}{\left\lfloor (N-m)/d\right\rfloor d}\right\}}{d}$$

If the average curve length < *L*_*m*_(*d*) >_*m*_ over *d* sets follows a power law, according to Equation (4), the time series has scaling properties, with a fractal dimension *FD*_*Hig*_.

(4) $$<L(d)>\propto d^{-FD_{Hig}}$$

The routine used in the estimation of Higuchi fractal dimension FD is available at https://uk.mathworks.com/matlabcentral/fileexchange/50290-higuchi-and-katz-fractal-dimension-measures.

#### 2.2.2. Detrendred Fluctuation Analysis

The Detrended Fluctuation Analysis (DFA) method is an alternative method (Peng et al., 1994, 1995), which estimates the Hurst exponent *H* in time series data instead of the fractal dimension.

The method consists of the following steps: Initially the time series with *N* time points *X*(1), *X*(2), …, *X*(*N*) is integrated as follows:

(5) $$y(k)=\overset{k}{\underset{i=1}{\sum^{\text{​}}}}(X(i)-\langle X\rangle )$$

Where *X*(*i*) represents the *i*−*th* element of the time series and < *X* > denotes the mean over the whole recording. The second step consists of dividing the time series into *N*_*l*_ windows of length *l*, then the mean square root of the integrated series is subtracted from the local trend, in every window (Peng et al., 1995), as shown in Equation (6).

(6) $$\begin{array}{c} \begin{array}{c} \begin{array}{c} \begin{array}{c} F(l)=\sqrt{\frac{1}{N_{l}}\overset{N_{l}}{\underset{k=1}{\sum^{\text{​}}}}{\left[y(k)-y_{l}(k)\right]}^{2}} \end{array} \end{array} \end{array} \end{array}$$

The local trend (*y*_*l*_(*k*)) is obtained from a linear regression over the time series in the window, and number *N*_*l*_ represents the total number of windows. In the following step, Equation (6) is obtained for several window lengths (*l*). The relation between *F*(*l*) and *l* is described by a power law, according to Equation (7), where *H* is the Hurst exponent.

(7) $$F(l)\propto l^{H}$$

The code used here is available in the Physionet repository (https://www.physionet.org/physiotools/dfa/) (Peng et al., 1995; Goldberger et al., 2000).

### 2.3. Multifractal Spectrum Estimation

In this section, we describe three multifractal spectrum estimators: Multifractal Detrended Moving Average (Gu and Zhou, 2010), Multifractal Detrended Fluctuation Analysis (Kantelhardt et al., 2002; Ihlen and Vereijken, 2010; Ihlen, 2012), and Chhabra-Jensen (Chhabra and Jensen, 1989), as these are the most established methods used in the literature.

**Figure 1:**
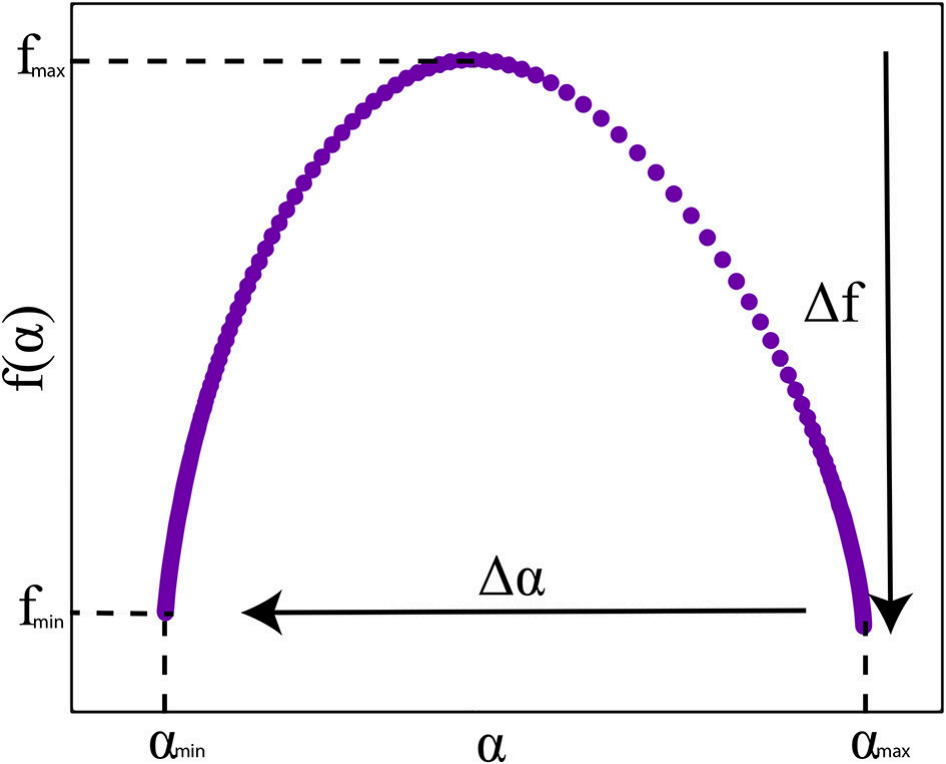
Multifractal singularity spectrum with a characteristic parabolic shape. The spectrum width (Δα) and height (Δ*f*) measures are indicated by the arrows.

Multifractal properties are represented as spectra (Figure 1), where essentially the fractal scaling properties, or more precisely Hausdorff dimensions [often noted as *f*(α)], are measured over a range of different singularities (α). Formally, the singularity spectrum is a function that describes the Hausdorff dimension of subsets of the time series *X*(*t*) with a specific Hölder exponent, according to:

(8) $$\begin{array}{c} f(\alpha )=D_{F}\{X(t_{s}),H(X(t_{s}))=\alpha \} \end{array}$$

Essentially, *f*(α) is the Hausdorff dimension (*D*_*F*_) of the subset (*t*_*s*_) of the time series *X*(*t*_*s*_) that has a the Hölder exponent α (van den Berg, 1999; Murcio et al., 2015). A definition of the Hausdorff dimension is available in Appendix D in Supplementary Material.

To characterize the function, or singularity spectrum *f*(α), usually, the width (Δα) and height (Δ*f*)—differences of maximum and minimum values of α and *f*(α), respectively—of the spectrum are used. Δα indicates the range of singularities present in a time series, this is also the most commonly used measure of how multifractal a time series is. The spectrum height Δ*f* indicates the range of Hausdorff dimensions present in the time series. See Figure 1 for an exemplary singularity spectrum plot.

#### 2.3.1. MF-DMA

Multifractal Detrended Moving Average (MF-DMA) is one of the most commonly used methods for the estimation of multifractal measures. The method of calculation consists of the following steps (Gu and Zhou, 2010): Given time series *X*(*t*) with time points *X*(1), *X*(2), …, *X*(*N*), the cumulative sum time series is obtained:

(9) $$\begin{array}{c} y(t)=\overset{N}{\underset{t=1}{\sum^{\text{​}}}}X(t) \end{array}$$

We then calculate the moving average over time windows of length *l*:

(10) $$\begin{array}{c} \tilde{y}(t)=\frac{1}{l}\overset{l-1}{\underset{z=0}{\sum^{\text{​}}}}y(t-z) \end{array}$$

A detrended version of the signal is obtained by the subtraction:

(11) $$\begin{array}{c} \epsilon (i)=y(i)-\tilde{y}(i) \end{array}$$

The resulting series is then divided in *N*_*l*_ disjoint sets of points of size *l* and a root-mean-square function is obtained for each set ν via:

(12) $$F_{\nu }(l)={\left\{\frac{1}{l}\overset{l}{\underset{i=1}{\sum^{\text{​}}}}\epsilon_{\nu }^{2}(i)\right\}}^{\frac{1}{2}}$$

A generalized *q*th-order overall fluctuation function can be obtained from:

(13) $$\begin{array}{c} F_{q}(l)={\left\{\frac{1}{N_{l}}\overset{N_{l}}{\underset{\nu =1}{\sum^{\text{​}}}}F_{\nu }{\left(l\right)}^{q}\right\}}^{\frac{1}{q}}\;q\neq 0 \end{array}$$

and

(14) $$\begin{array}{c} \ln F_{0}(l)=\frac{1}{N_{l}}\overset{N_{l}}{\underset{\nu =1}{\sum^{\text{​}}}}\ln\;F_{\nu }(l)\text{ for }q=0 \end{array}$$

It is possible to find a power-law relationship between *F*^*q*^(*l*) and the window length, or scale *l* by:

(15) $$F^{q}(l)\propto l^{\alpha (q)}$$

The multifractal “mass exponent” (Biswas and Cresswell, 2012) can be defined as:

(16) $$\begin{array}{c} \tau (q)=q\alpha (q)-D_{f} \end{array}$$

where *D*_*f*_ is the fractal dimension of the support measure. For a single-channel time series, *D*_*f*_ = 1. The spectrum, *f*(α), can be obtained with a Legendre transform (Gu and Zhou, 2010):

(17) $$\begin{array}{c} \alpha (q)=\frac{d\tau (q)}{dq} \end{array}$$

(18) $$\begin{array}{c} f(q)=q\alpha -\tau (q) \end{array}$$

It is important to note that the Legendre transform is known to cause problems in multifractal spectra derivations if some heterogeneities are present in the signal, as has been reported elsewhere (Chhabra and Jensen, 1989; Mukli et al., 2015).

#### 2.3.2. MF-DFA

The Multifractal Detrended Fluctuation Analysis (MF-DFA) method is essentially a generalization of the DFA approach (Kantelhardt et al., 2002; Ihlen, 2012). The time series is first rebuilt according to Equation (5).

It is then divided into $N_{l}=\frac{N}{l}$ non-overlapping epochs ν of length *l*. The variance of the detrended series is calculated as follows:

(19) $$F_{\nu }^{2}(l)=\frac{1}{l}\overset{n}{\underset{k=1}{\sum^{\text{​}}}}{\left(y((\nu -1)l+1)-y_{v}(k)\right)}^{2}$$

where *y*_ν_ represents the fitting in the epoch ν obtained via linear regression. The overall q-th order fluctuation functions can be obtained as:

(20) $$F_{q}(l)={\left\{\frac{1}{N_{l}}\overset{N_{l}}{\underset{\nu =1}{\sum^{\text{​}}}}{\left(F_{\nu }^{2}(l)\right)}^{\frac{q}{2}}\right\}}^{\frac{1}{q}}$$

A log-log plot of *F*_*q*_(*l*) vs. *l* for different values of q should present a linear curve defined by the power law in Equation (15). Similarly to the MF-DMA method, the multifractal scaling exponent can be defined as in Equation (16) and the spectrum *f*(α) can be determined in the same way as in the MF-DMA approach.

#### 2.3.3. Chhabra-Jensen

Multifractal spectra can be obtained in a more direct way, without the need for the Legendre transform using the Chhabra-Jensen (CJ) method (Chhabra and Jensen, 1989; Miranda et al., 2006; Zeleke and Si, 2006; Vázquez et al., 2008; Paz-Ferreiro et al., 2010a,b; Murcio et al., 2015; Xu et al., 2017; França et al., 2019). Considering a time series as a distribution over time, the approach consists of calculating a family of generalized measures by covering the time series with windows. These are probabilistic measures with an emphasis factor *q* that accentuates different singularities depending on its value. More singular regions are emphasized by *q*>1 whereas less singular regions will have a higher weight with *q* < 1 (Chhabra and Jensen, 1989).

First, we define:

(21) $$\begin{array}{c} \mu_{i}(q,l)=\frac{P_{i}{\left(l\right)}^{q}}{\sum_{j}P_{j}{\left(l\right)}^{q}} \end{array}$$

where *P*_*i*_(*l*) represents the cumulative probability of a window *i*. *l* corresponds to the size of the window in which the generalized measures are obtained. The window epochs are indexed by the variables *i* and *j*. Then the multifractal spectra can be obtained directly from:

(22) $$\begin{array}{c} \alpha (q)=\underset{l\to 0}{\lim}\frac{\sum_{i}\mu_{i}(q,l)\log P_{i}(l)}{\log l} \end{array}$$

and

(23) $$\begin{array}{c} f(q)=\underset{l\to 0}{\lim}\frac{\sum_{i}\mu_{i}(q,l)\log\mu_{i}(q,l)}{\log l} \end{array}$$

A numerical approximation to the equations above is provided by the measures *Mα* and *Mf* functions in Equations (24) and (25).

(24) $$\begin{array}{c} M\alpha =\underset{i}{\sum }\mu_{i}(q,l)\log P_{i}(l) \end{array}$$

(25) $$\begin{array}{c} Mf=\underset{i}{\sum }\mu_{i}(q,l)\log\mu_{i}(q,l) \end{array}$$

α and *f*(*q*) can then be obtained as the slopes by regressing these two measures against the scales *l*: *Mα*~*l* and *Mf*~*l*.

The algorithmic summary of the Chhabra-Jensen method consists of the following steps:

- The algorithm has as input the time series, a range of *q* values to which the spectrum will be evaluated, and window sizes *l* that vary in a dyadic scale.
- The time series is divided into non-overlapping epochs of length *l* and the generalized measures are estimated according to Equation (21).
- The measures *Mα* and *Mf* are obtained from the generalized measures.
- α and *f*(*q*) in Equations (22) and (23), respectively, are obtained with a linear regression procedure: log(*Mα*) is regressed against −log(*l*) and *log*(*Mf*) is regressed against −log(*l*), they give α and *f* respectively as the slopes.
- A rejection criterion is also used, where all *q* exponent values with *R*^2^ < 0.9 in the linear regression are not considered.

The code used in this study to calculate the multifractal spectrum is available at: https://github.com/lucasfr/chhabra-jensen. A flow-chart diagram of the algorithm is included in the repository above and in Appendix Figure E1 in Supplementary Material.

### 2.4. Data

#### 2.4.1. Simulating Fractal Time Series: Modulated Fractional Brownian Motion

**Figure 2:**
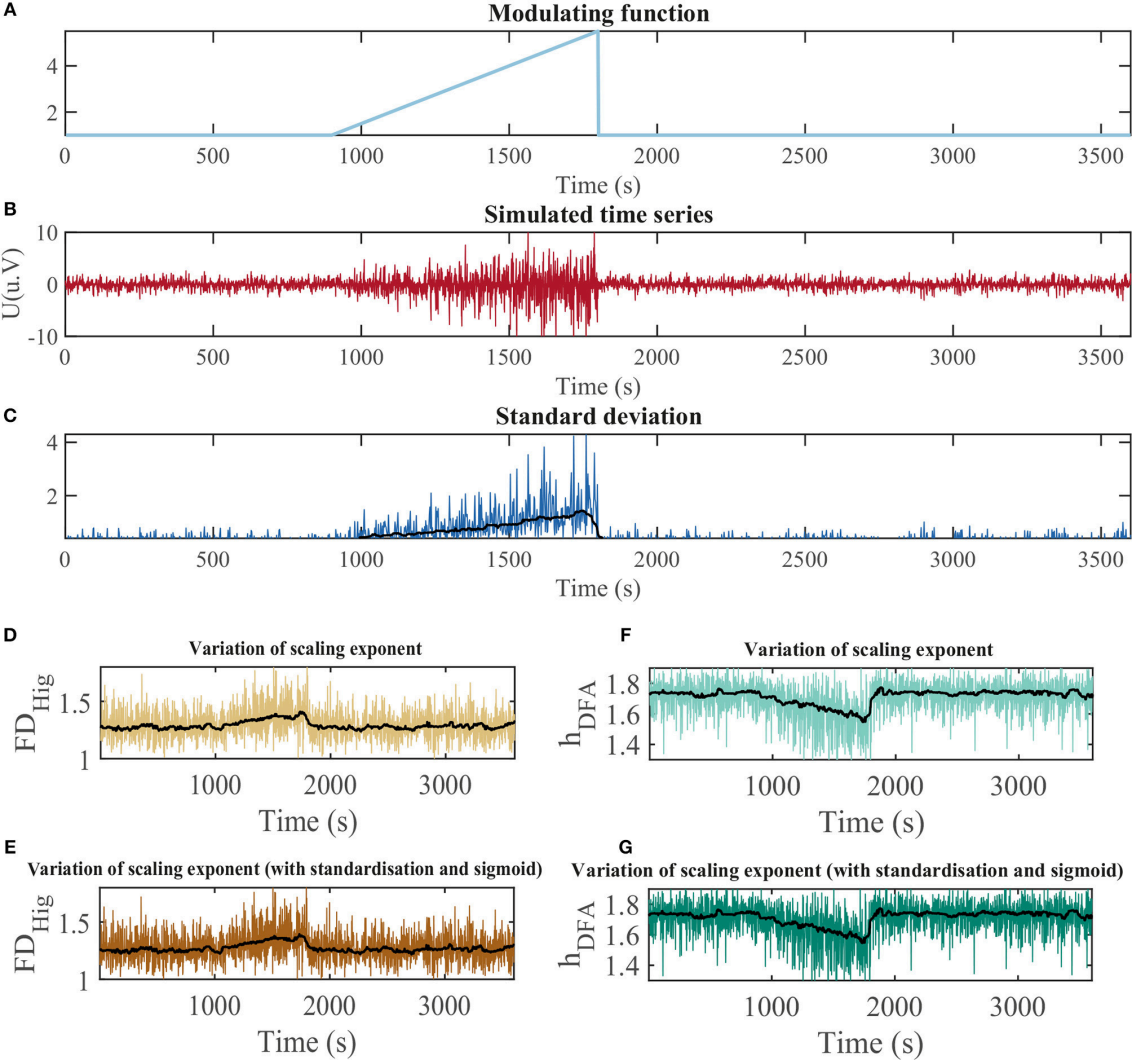
Impact of the signal standard deviation on monofractal scaling exponent estimation. **(A)** Modulation of the standard deviation of the time series over time; **(B)** Time series simulated using fractional Brownian motion based on modulation in **(A)**; **(C)** Standard deviation of the simulated signal in **(B)**. **(D)** Monofractal dimension obtained with the Higuchi method from signal without epoch-based standardization. **(E)** Monofractal dimension obtained with the Higuchi method from epoch-based standardized signal. **(F)** Hurst exponent obtained with the DFA method from signal without epoch-based standardization. **(G)** Hurst exponent obtained with the DFA method from epoch-based standardized signal.

To fully test methods of estimating the monofractal dimension from time series, we computationally produced time series that are known to be fractal (used for Experiment 1). We generated fractional Brownian motion (fBm) (Mandelbrot and Van Ness, 1968) profiles/time series using a novel modified version of the Wood-Chan or circulant embedding approach (Kroese and Botev, 2015; Shevchenko, 2015) that allow us to change the variance of the signal over time, in order to evaluate its influence on the fractal estimation. Our modulated fBM approach uses a modulating function, *M*(*t*), which produces a signal that has an amplitude varying over time. The details of fBm and our Modulated fBm (ModfBm) are described in Appendix A in Supplementary Material. The fBm time series was simulated with Hurst exponent *H* = 0.7; the value was chosen due to its persistent features, i.e., it generates a time series with memory. The modulating function *M*(*t*) used to modify the variance of the signal over time (see also described in Appendix A in Supplementary Material) is shown in Figure 2C. Using this method, we generated time series to evaluate the impact of variance change on monofractal estimators.

Note that there are alternative methods to generate monofractal time series (Davies and Harte, 1987; Eke et al., 2002; Mukli et al., 2015; Nagy et al., 2017). However, as our aim was not to compare generative models of monofractal time series, but rather simply demonstrate that the effects we observe in EEG signals could be more general. We chose the above mentioned approaches as example demonstrations.

#### 2.4.2. Simulating Multifractal Time Series: p-Model

Similarly to the fBm, we also used a computational procedure to generate time series that are known to be multifractal (for Experiment 2) based on the *p-model*, which was developed to reproduce features observed in turbulence experiments known to have multifractal properties (Meneveau and Sreenivasan, 1987). This is a simple model, having a single fraction *p*_1_ as its only input and is often mentioned in literature (Meneveau and Sreenivasan, 1987, 1991; Lipa and Buschbeck, 1989; She and Leveque, 1994; Consolini et al., 1996; Davis et al., 1997; Sreenivasan and Antonia, 1997; Kestener and Arneodo, 2003; Zhou, 2008; Pechlivanidis and Arheimer, 2015). Briefly the algorithm works as follows: From an interval of length *L* and height ϵ_*L*_ = *c* (is a constant), we create two segments of length *L*/2. Based on the input parameter *p*_1_, it is possible to establish a second fraction in which a second parameter will be given by *p*_2_ = 1−*p*_1_. The heights of each interval will thus be given by *y* = 2*p*_1_ϵ_*L*_, and *y* = 2*p*_2_ϵ_*L*_, respectively. This procedure is repeated for each remaining segment, selecting left or right for *p*_1_ randomly (Meneveau and Sreenivasan, 1987).

We employed the *p-model* in the simulation of a time series profile with multifractal properties to be evaluated by different estimation methods. It was generated with a code available at http://www2.meteo.uni-bonn.de/staff/venema/themes/surrogates/pmodel/ (Davis et al., 1997; Venema et al., 2006). Using this algorithm, we generated time series to evaluate the performance of different multifractal estimators with *p* = 0.4. The value was rounded (for simplicity) from the figure used elsewhere (*p* = 0.375) (Davis et al., 1997).

#### 2.4.3. Human EEG Data

Intracranial EEG data segments extracted from recordings in patients undergoing evaluation for epilepsy surgery were used for Experiments 3 and 4. In order to evaluate the effect of EEG signal variance change on multifractal properties (Experiment 3), we specifically looked for one recording, where the signal variance changes dramatically over time. One such recording was found in one patient (male, 28 years old, temporal lobe epilepsy, recorded at the National Hospital for Neurology and Neurosurgery (NHNN) (UCLH NHS Foundation Trust, Queen Square, London, UK), patient ID: “NHNN1”) near one seizure event. We used a 60-min recording segment around the epileptic seizure for our analysis. The seizure onset and offset were marked by expert clinicians, independent of this research project. Note that we used this segment specifically due to the dramatic change in signal variance, which actually occurred before the seizure and evolves over about 15 min. We do not make conclusions about the seizure event itself at this stage, but rather use this recording as an example to illustrate a technical point about multifractal property estimation from EEG.

To analyse the possible changes in multifractal properties during seizures (Experiment 4), we used a different dataset: Intracranial EEG from four subjects were retrieved from the ieeg.org repository (http://www.ieeg.org/) (Wagenaar et al., 2013): “I001_P005_D01,” “I001_P034_D01,” “I001_P010_D01,” and “Study 040.” These subjects were chosen due to the high sampling rate of their recordings (5 kHz), as we evaluated the impact of sampling frequency on multifractal properties. We extracted a 15-min segment around every seizure in each patient for further analysis. In Experiment 4, we performed the multifractal analysis on channels that were marked as seizure onset channels. We show the results for one patient in the main figure and the results for the remaining three patients are shown in Appendix B in Supplementary Material. Further information on the recordings is available in Appendix F in Supplementary Material.

The anonymized data analyzed in this study were recorded in patients undergoing evaluation for epilepsy surgery. iEEG.org portal provided EEG data and ethical approval for analyzing the data was provided by Mayo Clinic IRB (Brinkmann et al., 2009, 2016).

For NHNN data, the subject gave informed written consent, and the study was approved by the Joint Research Ethics Committee of the NHNN (UCLH NHS Foundation Trust) and UCL Queen Square Institute of Neurology, Queen Square, London, UK.

### 2.5. Pre-processing and Analysis of Time Series

Unless stated otherwise, we have applied the same pre-processing and analysis parameters to the computationally generated time series and the human EEG recordings and performed the fractal and multifractal estimations on 1,024-sample epochs. In Experiments 1 and 2, we were specifically interested in the effect of signal variance on the (multi) fractal estimation, and for comparison we also subjected the EEG signal to a standardization procedure, as follows:

(26) $$\begin{array}{c} x^{\prime }=\frac{X-<X>}{s} \end{array}$$

where < *X* > is the epoch mean and *s* the epoch standard deviation of the time series *X*, resulting in a time series with zero mean and unit standard deviation in each epoch.

The Chhabra-Jensen method requires as input a distribution function over the domain of positive real numbers, which is incompatible with EEG data which contain positive and negative values. Hence, we propose the use of a sigmoid-transformation here (Equation 27) to map the time series onto positive values, in order to apply the Chhabra-Jensen method. Example sigmoid functions and correspondingly transformed EEG signal are shown in Appendix Figure E2 in Supplementary Material.

(27) $$\begin{array}{c} \sigma (X)=\frac{1}{1+e^{vX}} \end{array}$$

The parameter *v* was chosen based on its effect on the estimated multifractal width for three types of time series: icEEG (NHNN1-channel 1), surrogate EEG (temporally shuffled values of the original time series from NHNN1-channel 1) and a simulated random series (with the same mean and variance), across the range *v* = [0.1, 2.0] in steps of 0.1. To find the optimal value for the parameter *v*, we needed to balance the trade-off between the three series in terms of presenting the most distinct Δα values (Appendix Figure E3A in Supplementary Material), while showing minimum distortion on the recording, or maximum correlation with the original time series (Appendix Figure E3B in Supplementary Material). We chose *v* = 1 as an acceptable trade-off point. Finally, to compare multifractal properties to classical EEG frequency band power, we used the following definitions for the classical EEG frequency bands: δ (0.5–4 Hz), θ (4–8 Hz), α (8–15 Hz), β (15–30 Hz), and γ (30–60 Hz).

## 3. Results

### 3.1. Experiment 1: Monofractal Estimation With Respect to Changing Signal Variance

We evaluated the relationship between monofractal measures and signal variance using a simulated time series based on fractional Brownian motion (fBm), where its signal variance is modulated by a modified ramp function. The modulation function is shown in Figure 2A and resulting time series in Figure 2B. The standard deviation of the generated time series indeed tracks the shape of the modulating function (Figure 2C).

We estimated the monofractal dimension of this simulated signal using two standard methods: Higuchi and DFA. We observe that both methods appear to be affected by the changing signal variance (Figures 2D,F). Furthermore, the effect persists even after epoch-based standardization (Figures 2E,G): the monofractal properties and standard deviation correlate with ρ = 1.00 and ρ = 0.99 for the Higuchi and DFA methods, respectively. A similar effect was observed for a real icEEG recording that contained changes in signal variance over time (Appendix Figure E4 in Supplementary Material).

In conclusion, monofractal properties derived for each epoch from DFA and Higuchi methods (with, or without signal standardization) correlate highly with the signal standard deviation of the epoch. Therefore, in epoch-based approaches (e.g., for application such as detecting or predicting epileptic seizures), the monofractal properties cannot be regarded as a new useful EEG feature of an epoch that is not redundant to standard deviation of the epoch. Thus we turn our attention to multifractal properties of the signal next.

### 3.2. Experiment 2: Multifractal Estimation Stability

In the following, we will denote the epoch-wise estimates of multifractal width Δα and height Δ*f* (and Δα^†^ and Δ*f*^†^ for the measure of the epoch-based standardized time series).

This experiment was designed to assess the reliability of the different multifractal estimation methods over time. In other words, if the multifractal properties of the time series remain constant over different epochs, then we expect the multifractal estimation method to show the same output over these different epochs. Note that the accuracy of these methods (i.e., the method outputting the expected multifractal measures of a predefined multifractal object with known multifractal properties) has been demonstrated elsewhere (Chhabra and Jensen, 1989; Kantelhardt et al., 2002; Gu and Zhou, 2010).

**Figure 3:**
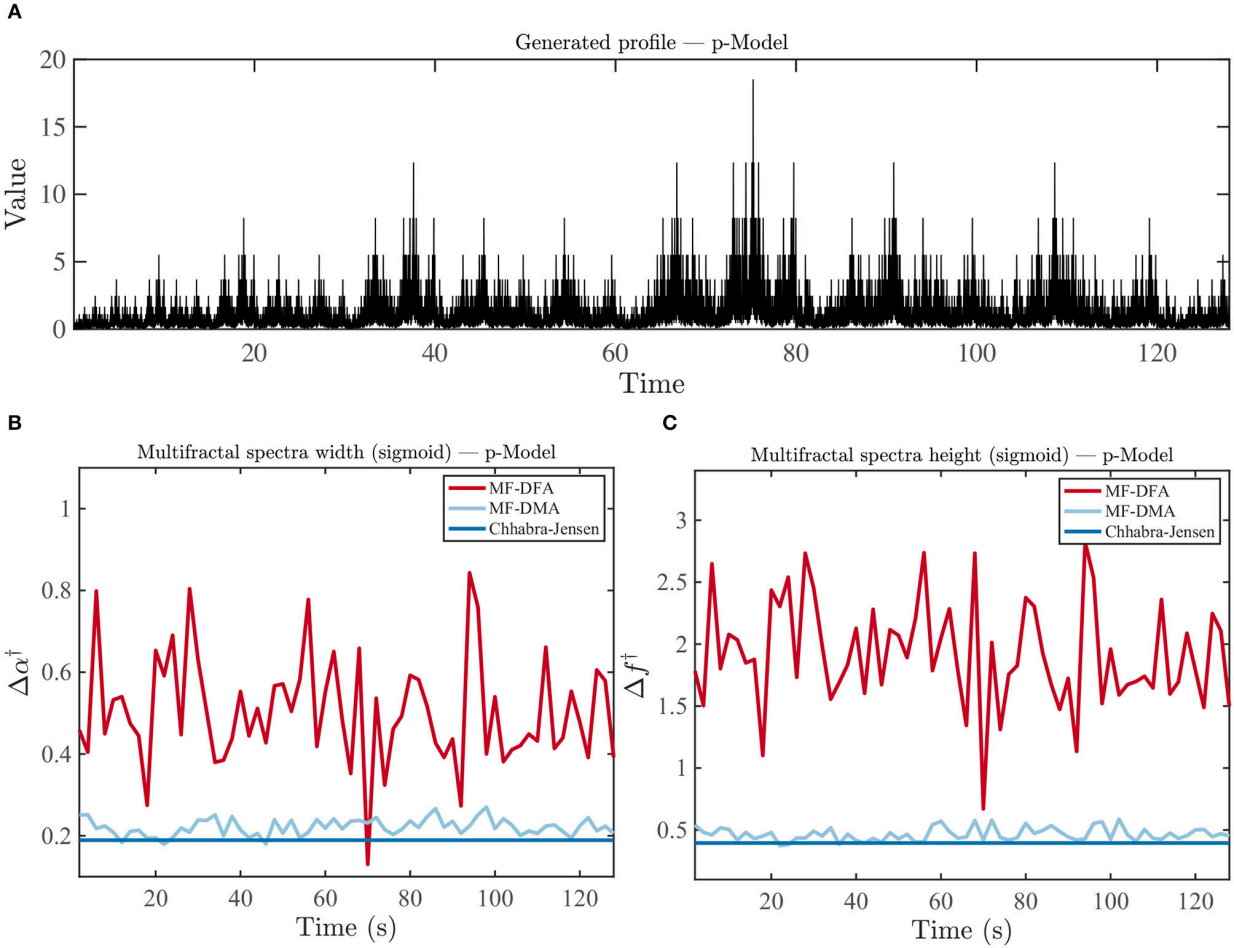
Comparison of three multifractal spectrum estimation methods (MF-DFA, MF-DMA, and Chhabra-Jensen) for p-Model simulated time series. **(A)** Time series simulated for *p* = 0.4. **(B)** Estimated multifractal spectra width Δα^†^ and **(C)** height Δ*f*^†^.

Figure 3 shows the simulated signal by the p-model and the outputs of the three multifractal spectral estimation methods. In all cases, the magnitude of Δα^†^ and Δ*f*^†^ were clearly different from zero. The (Δα^†^, Δ*f*^†^) output variances over time for the MF-DFA, MF-DMA, and Chhabra-Jensen estimation methods were: (0.018, 0.18), (4.17e-4, 0.0028), and (2.3e-30, 6.5e-30), respectively. In addition, the MF-DFA output violated the theoretical topological limit of Δ*f*^†^ = 1, again indicating problems in the MF-DFA method, potentially due to the inversion of multifractal spectrum (Mukli et al., 2015). As the Chhabra-Jensen method shows the lowest variance over time (i.e., most reliable/stable), it will be our multifractal analysis method of choice for the remainder of this work.

### 3.3. Experiment 3: Multifractal Estimation of Human EEG and Its Potential Added Value

**Figure 4:**
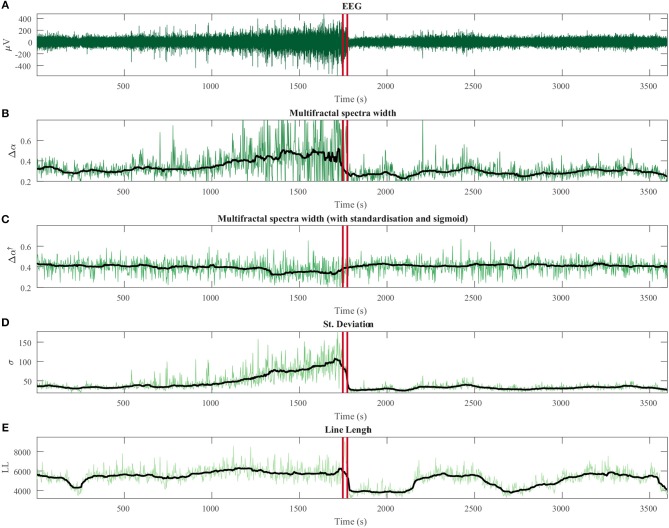
Temporal dynamics of multifractal spectrum width compared with conventional measures for human intracranial EEG. **(A)** Intracranial EEG segment containing a seizure (onset and offset marked with red vertical lines). Note that this recording was chosen because it showed a dramatic change in signal variance during non-seizure periods, not because of any seizure related properties. **(B)** Variation of multifractal spectrum width without epoch-wise standardization (Δα). **(C)** Multifractal spectrum width based on epoch-wise standardized time series (Δα^†^). **(D)** Standard deviation in each epoch. **(E)** Line length in each epoch. Black line: moving average of each measure.

Next, we evaluated the relationship between multifractal signal properties and other widely used conventional EEG measures (such as signal variance). Figure 4 shows the results of the multifractal spectrum and conventional measures in comparison. The pattern of multifractal spectrum width without epoch-based standardization (Δα) reflects the signal variance closely, in contrast to the estimate for the epoch-based standardized signal (Δα^†^). Finally, signal line length also shows a very different temporal profile from Δα^†^. A similar figure showing the variation of Δ*f* and Δ*f*^†^ metrics is available in Appendix Figure E5 in Supplementary Material.

**Figure 5:**
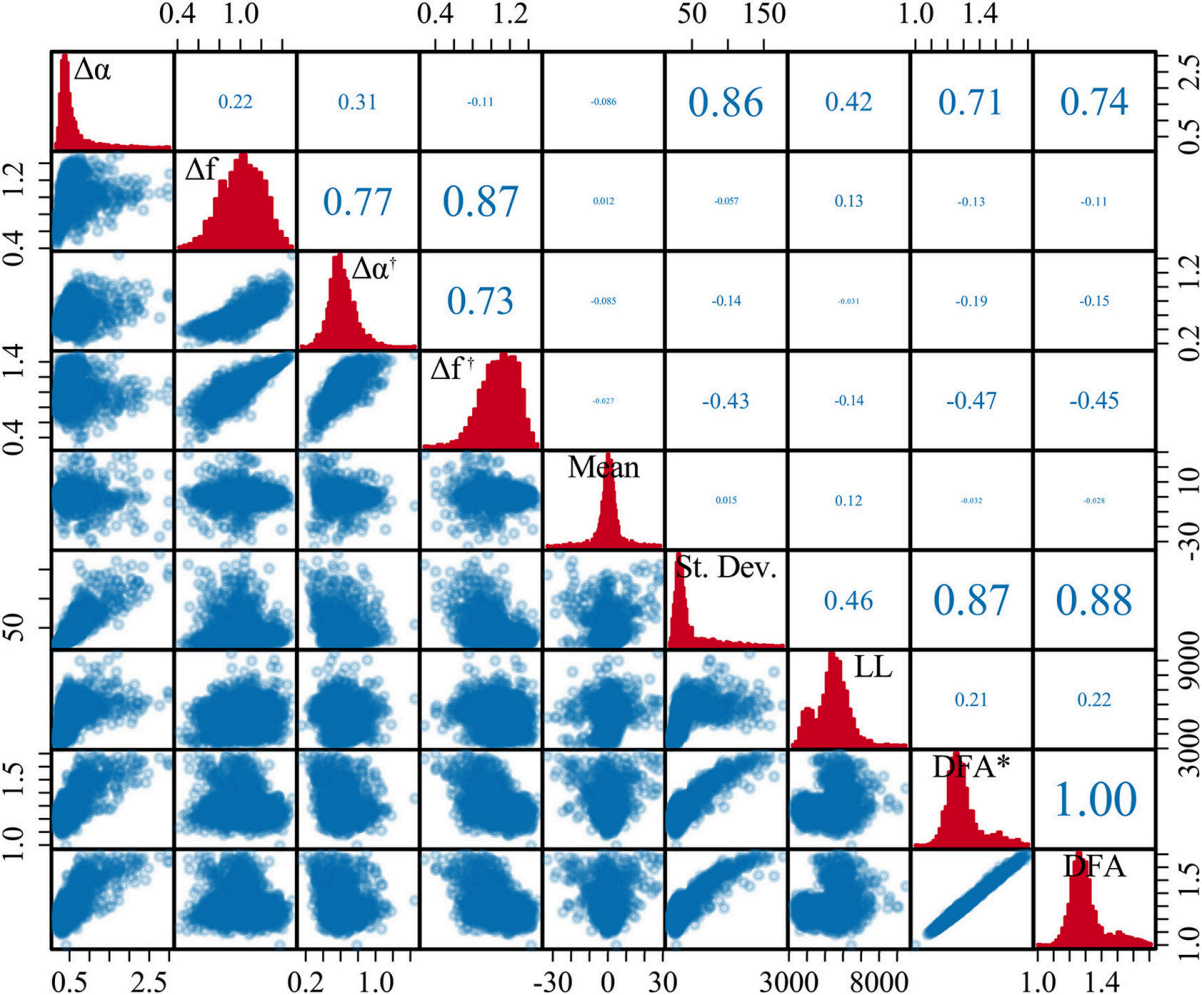
Correlation between multifractal spectrum and conventional EEG measures for human icEEG data (from Figure 4). The diagonal of the matrix shows the distribution for each measure across epochs. The lower triangle contains the scatter plots for each pair of measures across epochs. The upper triangle shows the Pearson correlation value for each pair of measure, where the size of the font additionally corresponds to the correlation coefficient to provide an additional visual cue.

Figure 5 shows the quantification of similarities of the signals in Figure 4 through a correlation analysis. In summary, a high degree of correlation is present between the signal standard deviation, multifractal spectrum width (Δα), and detrended fluctuation analysis (monofractal approach) both with and without epoch-based standardization. We found that standardization reduces the correlation between Δα and the standard variation from ρ = 0.86 (for Δα ) to ρ = −0.14 (for Δα^†^). We also note that Δα is highly correlated with DFA and DFA^†^ estimates (ρ = 0.74 and ρ = 0.71, respectively) while it is markedly reduced for Δα^†^ (|ρ| < 0.3). The analysis based on the mutual information (Ince et al., 2017) rather than correlation showed a similar pattern (Appendix Figure E6 in Supplementary Material).

**Figure 6:**
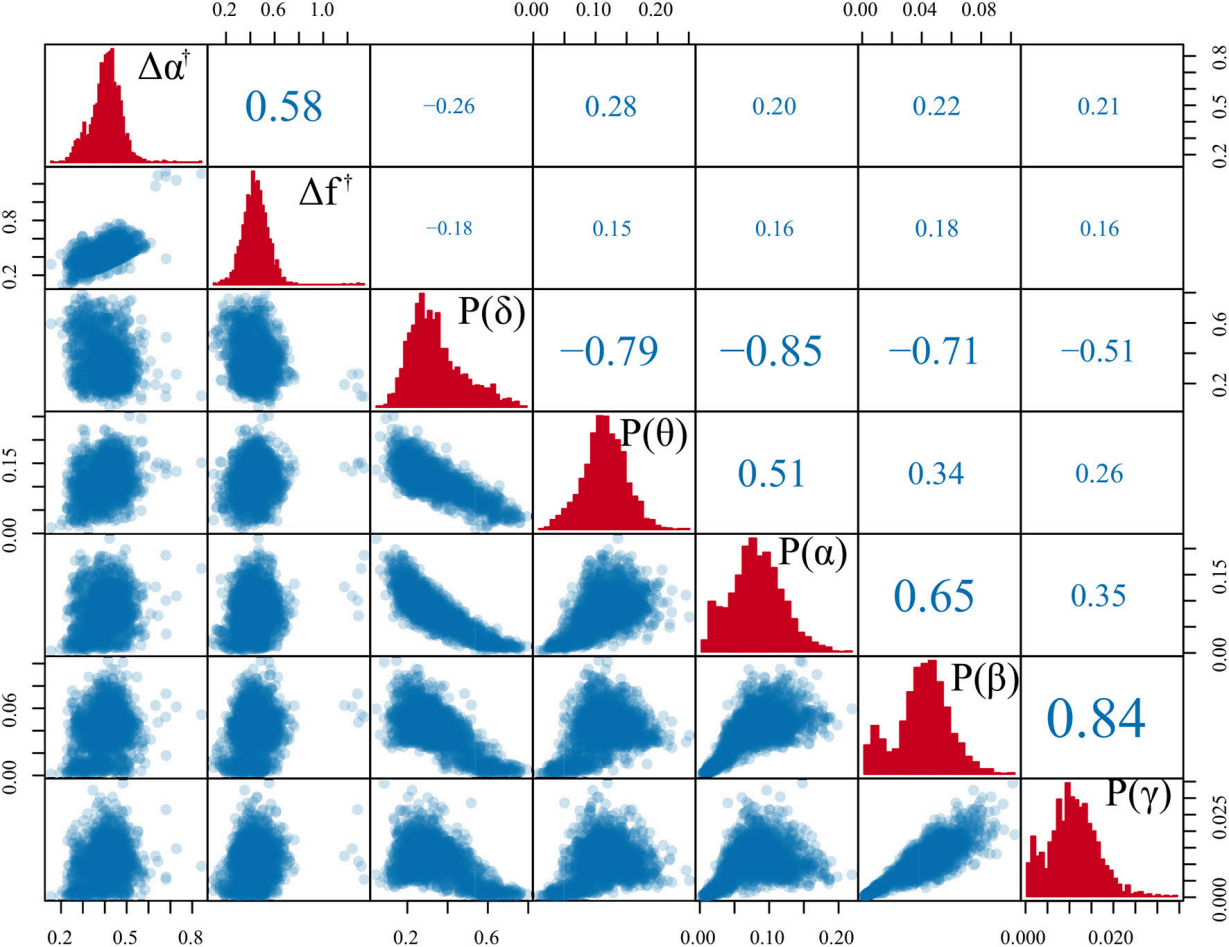
Comparison of multifractal measures with classical spectral band power. Scatter plot matrix comparing both standardized multifractal spectrum width and height (Δα^†^ and Δ*f*^†^) with the δ, θ, α, β, and γ average band power in each epoch. Each scatter point is derived from a single epoch of the time series. The diagonal of the matrix features the histograms for each measure. The lower triangle contains the scatter plots for each pair of measures. The upper triangle shows the Pearson correlation for each pair of measure, where the size of the font additionally corresponds to the correlation coefficient to provide an additional visual cue. The icEEG data underlying this figure is shown in Figure 4A.

The relationships of the multifractal properties and specific EEG frequency band power are shown in Figure 6. In summary, the correlation values between the multifractal measures Δα^†^, Δ*f*^†^, and signal power in the classical EEG bands are low (|ρ| < 0.3). A supplementary analysis of EEG time series data containing different sleep stages (which are known to be dominated by specific frequencies) shows similar results (see Appendix C in Supplementary Material). Based on these results, we focused on Δα^†^ (using epoch-wise standardization of the time series) in the subsequent analysis.

### 3.4. Experiment 4: Impact of Sampling Frequency and Epoch Length on Multifractal Estimation of Human EEG

**Figure 7:**
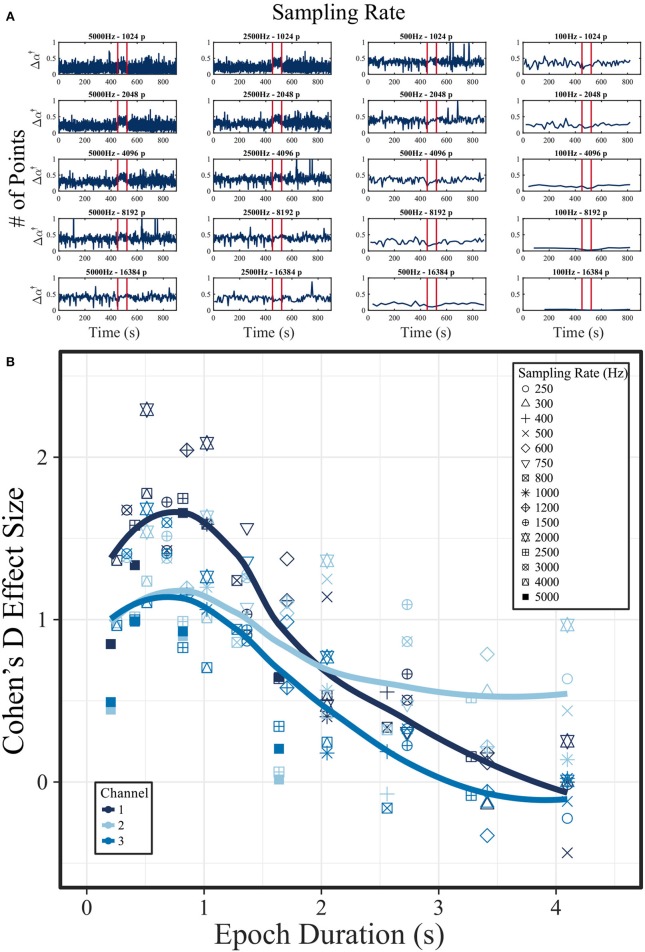
Influence of EEG sampling frequency and epoch length on multifractal spectrum width around and during an epileptic seizure. **(A)** Multifractal spectrum width (Δα^†^) in a 15-min intracranial EEG segment containing one seizure (onset and offset marked by the red lines). The signal was initially sampled at 5,000 Hz. Each column shows Δα^†^ for 5,000, 2,500, 500, and 100 Hz sampling rates. Different epoch sizes were used ranging from 1,024 to 16,384 samples (in each row). **(B)** Relationship of effect size *D* (between the interictal and ictal distribution of Δα^†^) and epoch duration in seconds (obtained by dividing the number of sampling points by the sampling rate of the signal). Channel 1 is the data shown in **(A)**. The solid line represents a LOESS curve fitting of the data points, with formula “y ~ x.” The data used for this figure is obtained from for subject “I001_P005_D01” around seizure 1. Channel 1: ADMacro_01. Channel 2: ADMacro_02. Channel 3: ADMacro_03.

The variation of the multifractal spectrum width Δα^†^ for different combinations of epoch sizes and sampling frequencies is shown in Figure 7A. On visual inspection, it is clear that there are some combinations of epoch size and sampling frequency that show a clear increase of Δα^†^ during the ictal period (marked by the red lines). To quantify this effect, Figure 7B shows the Cohen's effect size *D* of the ictal vs. interictal Δα^†^ distributions plotted against epoch duration (in seconds). In this plot, we included 15 different sampling frequencies, and also data from three different EEG channels (all in the seizure onset zone). A peak in *D* can be seen at about 1 s (across all sampling frequencies), indicating that the change in Δα^†^ during a seizure can be best captured when using 1 s epochs (regardless of sampling frequency). This effect was not found for the sampling frequency or epoch length separately. Similar results for additional patients are shown on Supplementary materials (Appendix B in Supplementary Material).

## 4. Discussion

In this study, we have explored the monofractal and multifractal properties of human EEG recordings and used simulated data to test the performance of fractal property estimation methods. Although mono- and multi-fractal approaches have been widely employed in the study of physiological signals in humans (Ivanov et al., 1999; Stanley et al., 1999; Hu et al., 2004, 2009; Costa et al., 2017; França et al., 2019), we have demonstrated that the monofractal dimension may be capturing a similar signal feature as the signal variance. When using standardization to remove the effect of signal variance, we demonstrated that multifractal measures (estimated by the Chhabra-Jensen method) capture information not contained in widely used conventional signal measures, making it a viable feature for machine learning in clinical EEG applications. Finally, using epileptic seizure as an example, we showed that the epoch length can significantly impact the detection of time-varying effects in multifractal properties, suggesting the need for data- and application-specific optimization.

### 4.1. Methodological Considerations

One of our key observations is that monofractal estimators are tightly correlated with signal variance—even following epoch-wise standardization, whereas multifractal properties following epoch-wise standardization are no longer tightly correlated with signal variance. This may appear to be a curious and non-intuitive observation that, to our knowledge, has not been reported before.

To interpret this observation, it is worth noting the relationship between monofractal and multifractal analyses. Essentially, in multifractal analysis, at the point for which *q* = 2, the corresponding *f*(α) is the so-called correlation dimension, which is an alternative way of estimating the monofractal dimension (Murcio et al., 2015). The relationship between monofractal dimension and signal variance has been established and explained before (Cannon et al., 1997). By the same token, signal variance also affects higher statistical moments (*q*>2 or *q* < −2). However, when analysing the exact effect of variance on the multifractal spectrum (Appendix Figure E9 in Supplementary Material), we observe that the variance particularly impacts the multifractal spectrum width and height, but maintains an almost constant value of *f*(α) for *q* = 2. This explains why epoch-wise standardization does not impact monofractal dimension but does impact multifractal spectrum width and height. The mono- and multifractal properties we are investigating here are essentially describing different properties of the multifractal spectrum. Note that through our standardization procedure, we do not abolish “multifractality,” but only its dependence on signal variance. Future work has to show mathematically the exact reason for this observation, although intuitively it is understandable that the standardization procedure (a linear transformation of the signal) changes the *q* = 2 moment least and affects higher moment more.

We further observed that the Chhabra-Jensen method is the most reliable out of the three multifractal estimation methods. As was pointed out in the original publication (Chhabra and Jensen, 1989), this is most likely due to the fact that the Chhabra-Jensen method avoids a Legendre transform that the other methods require. The Legendre transformation requires smoothing of the *D*_*q*_ curve and can lead to errors. For further advantages of the Chhabra-Jensen method, the reader is referred to the original publication (Chhabra and Jensen, 1989). A recent development, FMF method (Mukli et al., 2015; Nagy et al., 2017), may be an alternative to the approach proposed in this study.

Finally, our analysis highlighted the importance of choosing an adequate epoch size given a sampling frequency, in order to study events such as epileptic seizures. However, our study was based on the analysis of ictal vs. interictal epochs, i.e., a hard separation that may not represent continuous phenomena accurately. Future work should take into account that multifractal properties may be continuously changing over time (a striking example is shown in Appendix Figure E7 in Supplementary Material), and an explicitly time based approach may be needed. Along similar lines, our finding of a optimal time scale may be due to the non-stationary nature of the multifractal properties. Further theoretical work may have to develop a temporally resolved multifractal estimator, in order to fully understand this aspect.

### 4.2. Implications for the Understanding of Brain Activity and Brain Generators

Previous studies reported that the brain is characterized by critical dynamics (Eguíluz et al., 2005; Chialvo, 2010, 2012; Racz et al., 2018). This characteristic, found from microscopic spatial scales (such as neuronal networks) (Beggs and Plenz, 2003, 2004) to the whole-brain level (Eguíluz et al., 2005), is thought to facilitate the storage and processing of information. It has been further suggested that more than one scaling exponent would be necessary to properly characterize the brain's critical dynamics (Suckling et al., 2008; Ihlen and Vereijken, 2010; Ciuciu, 2012; Fraiman and Chialvo, 2012; Zorick and Mandelkern, 2013; Papo, 2014; Zhang et al., 2015; Papo et al., 2017; Racz et al., 2018), as departures from the power-law pattern have been frequently observed in brain signals. Hence, it has been proposed that using additional, higher-order statistical moments can better characterize such data (Fraiman and Chialvo, 2012). In this work, we contribute a complementary observation: while monofractal measures of EEG appeared to essentially follow the slow changes of signal variance, multifractal characterization is capable of revealing new information.

In terms of generative processes that can produce monofractal properties, it has been suggested that a property called Self-Organized Criticality (SOC) (Bak et al., 1987) may play an essential role. SOC describes the capacity of a system to evolve naturally into a critical state (a state in which a minimum perturbation could lead to events of all sizes). Such phenomena display power-law distributions and fractal properties as signatures (Bak and Paczuski, 1995). An example process that displays SOC is the so-called single avalanche or Bak–Tang–Wiesenfeld model (also known as Abelian sandpile model) (Bak et al., 1987). SOC behavior has been linked to physiological control mechanisms, such as in human heart rate variability (Goldberger et al., 2002). Similar to SOC, a related regime—termed non-classical SOC—is thought to give rise to multifractal properties (Lovejoy and Schertzer, 2007). The analysis and understanding of the non-classical SOC is, however, still under development.

In this context, our multifractal spectral analyses of human EEG data suggest that cerebral phenomena should not be modeled by a single avalanche model (classical SOC), in agreement with findings in a previous study (Fraiman and Chialvo, 2012). Moreover, it is hypothesized that brain dynamics are non-ergodic (Bianco et al., 2007), i.e., display preferential states and depends on previous states (Papo, 2014), which are all properties of multifractal processes (Lovejoy and Schertzer, 2007). Thus, multifractal analyses could provide a new paradigm for studying brain function and structure, as previously suggested in other studies of normal (Suckling et al., 2008; Ihlen and Vereijken, 2010; Ciuciu, 2012; Zorick and Mandelkern, 2013; Papo, 2014; Papo et al., 2017; Racz et al., 2018) and pathological brain activity (Zhang et al., 2015). Furthermore, generative processes displaying multifractal properties could help understanding the observed multifractal changes on a mechanistic level.

### 4.3. On the Detection of Brain State Transitions in Health and Disease

We want to emphasize that the conclusions from our work are drawn on the basis that slow changes in signal fractal features can be captured by using an epoch-wise feature extraction procedure. It is also from a feature redundancy perspective that we argue for the need of multifractal approaches over monofractal measures. We do not dispute the usefulness of monofractal measures in other general applications. In our work, we essentially performed a feature selection procedure using correlation and mutual information (Guyon and Elisseeff, 2003). We evaluated how different signal feature compare on an epoch-wise basis. Feature selection is crucial to obtain faster and cost effective models, and avoids overfitting of the available data. It might also help achieving a deeper insight into the nature of the studied phenomena (Blum and Langley, 1997; Liu et al., 1998; Guyon and Elisseeff, 2003; Liu and Yu, 2005; Saeys et al., 2007).

A fundamental observation in our work is that an optimal time scales may exist for specific physiological processes (such as epileptic seizures) in terms of their multifractal dynamics (Figure 7 and Appendix B in Supplementary Material). This result suggests that, at least in an epoch-based study, for any given epileptic seizure in a given patient, the variety of scaling exponents (Δα) will depend on the length of the epoch analyzed. This is further supported by similar findings in monofractal analysis (Eke et al., 2002). The implications of this observation are that certain scaling exponents will only exist in specific time scales and the diversity of scaling exponents will depend on the duration of the epoch. These results suggest the potential need for “tuning,” i.e., potentially having to find the characteristic time for every studied phenomenon. If this is indeed the case, a temporally resolved (not epoch-based) multifractal method should be developed in future to adequately characterize brain dynamics.

Furthermore, the slow temporal changes in multifractal dynamics need to be characterized in a systematic way. Using epileptic seizures as an example, Appendix Figure E7 in Supplementary Material shows that dramatic changes in multifractal properties can sometimes be seen before an epileptic seizure. This observation requires further investigation to address questions such as: are all epileptic seizures characterized by pre-ictal changes in multifractal properties? Do other physiological processes, such as sleep, influence this finding? To answer these questions, we will most likely also need well-characterized experimental conditions, where seizures can be triggered in a controlled manner.

Finally, it is well-recognized that epileptic seizures are spatio-temporal processes (see e.g., Wang et al., 2014, 2017), and our current approach of only focusing in the temporal aspect in one location will need to be expanded. Data-driven unsupervised approaches, such as dimensionality reduction, may help summarize spatial aspects. Additionally, the challenge will be to develop a spatio-temporal multifractal analysis approach that can also deal with the challenges of low spatial sampling resolution in EEG recordings.

### 4.4. Outlook

Our work has highlighted several challenges that need to be considered when analysing multifractal properties of EEG signals; namely choice of the appropriate estimation method, estimation parameters, and the influence of the time series variance on signal features. We have suggested some solutions to these problems, such as the used of the Chhabra-Jensen approach combined with an epoch-wise standardization approach, which has shown potential capabilities as a signal feature for machine learning applications. We have also highlighted possible process-specific challenges. In terms of epileptic seizures, future work is required to analyse a larger number of patients in order to draw firmer conclusions on the potential clinical relevance of multifractal analyses. Furthermore, the study of mechanistic generative models of EEG may shed light on why those multifractal changes occur. For example, a generative process of potential interest could feature a modified version of Bak–Tang–Wiesenfeld model (Bak et al., 1987).

### 4.5. Summary

In this paper, we have analyzed the monofractal and multifractal properties of human EEG recordings. We have shown that monofractal estimates are influenced by the standard deviation of the time series, thus not capturing features beyond signal variance. For multifractal estimation, we have shown that the Chhabra-Jensen approach is the most stable, and we have developed a method of signal pre-processing to remove the influence caused by the variance of the signal. Using the suggested approach, the multifractal estimates do not correlate with traditional EEG measures, thus yielding additional information about the signal and being a relevant signal feature. Finally, our results also indicate a preferential time scale to identify differences in multifractal properties between ictal and interictal state recordings in patients with epilepsy.

## Data Availability Statement

The datasets analyzed for this study can be found in https://github.com/yujiangwang/MultiFractalEEG.

## Author Contributions

LF, LL, MW, and YW: conceptualization, project administration, resources, and writing of the original draft. LF: data curation, funding acquisition, investigation, and visualization. LF and YW: formal analysis and validation. JM, LF, LL, MW, and YW: methodology. JM, LF, and YW: software. LL, MW, and YW: supervision. JM, LF, LL, MW, ML, NS, and YW: writing of the review, and editing.

### Conflict of Interest Statement

The authors declare that the research was conducted in the absence of any commercial or financial relationships that could be construed as a potential conflict of interest.
