# Supplementary material for: Fractal and Multifractal Properties of Electrographic Recordings of Human Brain Activity: Toward Its Use as a Signal Feature for Machine Learning in Clinical Applications
Source: Front Physiol. 2018 Dec 10;9:1767. doi: 10.3389/fphys.2018.01767 (PMC6295567; doi:10.3389/fphys.2018.01767)
Supplement: Supplementary file 1 [file Data_Sheet_1.pdf]

Appendix A: Fractional Brownian Motion

A fractional Brownian motion is a continuous zero-mean Gaussian process that can be described by the function in equation 2 [4]. In order to simulate such properties, it is sufficient to simulate the increments on the profile, as described in equation 1.

$$\xi_1 = B_1^H, \xi_2 = B_2^H - B_1^H, \xi_N = B_N^H - B_{N-1}^H \tag{1}$$

These values form a stationary sequence of standard Gaussian variables with a covariance described in equation 2. The vector $\xi = (\xi_1, ..., \xi_N)^T$, composed by the simulated increments, is called fractional Gaussian noise and is a centred Gaussian array with covariance matrix Ω [5].

$$\rho_H(n) = E[\xi_1 \xi_{n+1}] = \frac{1}{2}\bigg((n+1)^{2H} + (n-1)^{2H} - 2n^{2H}\bigg), \quad n \geq 1 \tag{2}$$

$$\Omega = \text{Cov}(\xi) = \begin{pmatrix} 1 & \rho_H(1) & \rho_H(2) & \cdots & \rho_H(N-2) & \rho_H(N-1) \\ \rho_H(1) & 1 & \rho_H(1) & \cdots & \rho_H(N-3) & \rho_H(N-2) \\ \rho_H(2) & \rho_H(1) & 1 & \cdots & \rho_H(N-4) & \rho_H(N-3) \\ \vdots & \vdots & \vdots & \ddots & \vdots & \vdots \\ \rho_H(N-2) & \rho_H(N-3) & \rho_H(N-4) & \cdots & 1 & \rho_H(1) \\ \rho_H(N-1) & \rho_H(N-2) & \rho_H(N-3) & \cdots & \rho_H(1) & 1 \end{pmatrix}$$

In order to solve this problem, we can, alternatively, convert the covariance matrix into a circulant matrix. The circular matrix (Σ) has a known eigenvalues structure, making it easier to solve the problem. Taking the relationship $M = 2(N-1)$, a circular matrix Σ is defined based on the relations defined by equation 3.

$$\begin{aligned} c_0 &= 1, \\ c_k &= \begin{cases} \rho_H(n), & n = 1, 2, ..., N-1 \\ \rho_H(M-n), & n = N, N+1, ..., M-1 \end{cases} \end{aligned} \tag{3}$$

$$\Sigma = circ(c_0, c_1, ..., c_{M-1}) = \begin{pmatrix} c_0 & c_1 & c_2 & \cdots & c_{M-2} & c_{M-1} \\ c_{M-1} & c_0 & c_1 & \cdots & c_{M-3} & c_{M-2} \\ c_{M-2} & c_{M-1} & c_0 & \cdots & c_{M-4} & c_{M-3} \\ \cdots & \cdots & \cdots & \ddots & \cdots & \cdots \\ c_2 & c_3 & c_4 & \cdots & c_0 & c_1 \\ c_1 & c_2 & c_3 & \cdots & c_{M-1} & c_0 \end{pmatrix}$$

We then seek for a factorisation described in the equation 4 [4]. The increments in the simulated Brownian motion time series will be derived from the eigenvalues $\lambda = (\lambda_1, ..., \lambda_{4N^2})^T$ arranged as a $2N$x$2N$ matrix Λ. The variable P is the Kronecker product of two discreet Fourier transform matrices given by $P = F \bigotimes F$ [4].

$$\Sigma = P^* \Lambda P \tag{4}$$

$$F_{jk} = \frac{1}{\sqrt{2N}} e^{-2\pi ijk/2N}, \quad \text{where} \quad j,k = 0, 1, ...2N-1 \tag{5}$$

The algorithm used to derive the fBm time series is available at `https://github.com/lucasfr/ModfBm` and can be summarised in the following steps: First, we compute the covariance matrix (Ω)(step 1) and build a $2N$x $2N$ circulant matrix (Σ) (step 2). For this operation,

we just need the first row of the matrix Σ (step 3). The eigenvalues of matrix Λ can then be obtained via Fourier transformation. Both real and complex parts will present a Gaussian profile, however, it is enough to consider the real part of the eigenvalues (step 4).
The following step consists of multiplying the output of step 4 by random complex numbers and applying the inverse Fourier transformation (step 5). We modified this step and added an element of a function M that will be multiplied by these complex random values before applying the inverse Fourier transformation. From now on, this modified version will be denoted as Modulated fractional Brownian motion (ModfBm)
The last step step consists of accumulating the values generated by the previous operation and multiplying by a constant scaled by the the Hurst exponent H. The generated time series should present scale properties according to the chosen H exponent and, for our ModfBm, feature changes on its standard deviation.

We generated a time series of 1.843.200 points (1800 windows of 1024 points), corresponding to a recording of an hour of duration with a sampling rate of 512 Hz - similar to the clinical intracranial EEG segment from the patient 'NHNN1'. The time series was simulated with a Hurst exponent $H = 0.7$, the value was chosen due to its persistent features, i.e., it generates a time series with memory. Additionally, a modulating function M, for every 2 seconds window w, described by 6 and shown in Fig. 3(C) was used to simulate the Modulated fBm.

$$M(w) = \begin{cases} 1, & w < 450 \\ 1 + (w - 449)0.01, & 450 \leq w \leq 900 \\ 1, & w > 900 \end{cases} \tag{6}$$

Appendix B: Characteristic time scale for other iEEG subjects

All the following datasets analysed are taken from the iEEG database (`https://www.ieeg.org/`). Study IDs are shown as on the database. The example channels we analysed were labelled seizure onset zone channels. The analysis method is the same as for the main text Fig. 7.

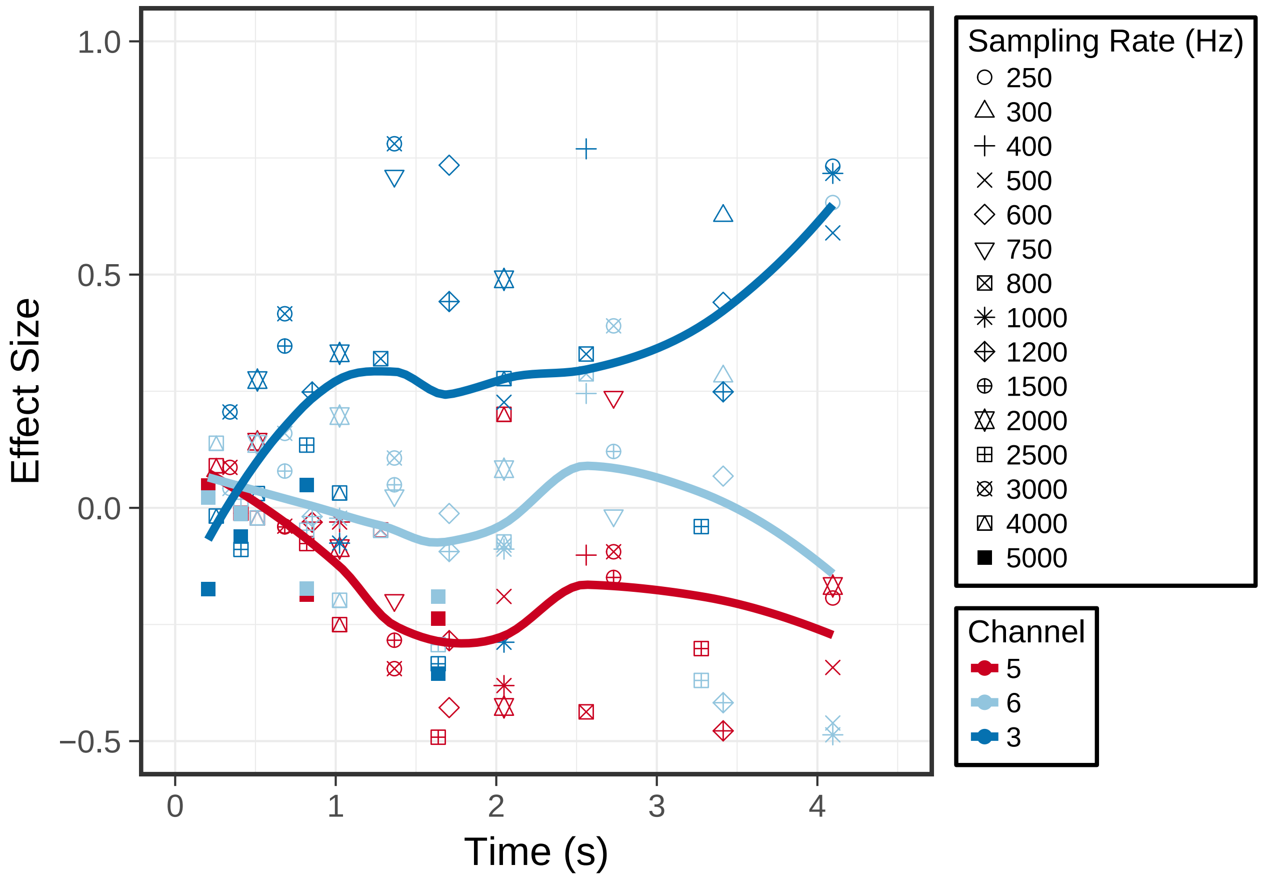

Figure 1: **I001_P010_D01. The three different channels are 'ITS_1','ITS_2', 'LG_08'.**

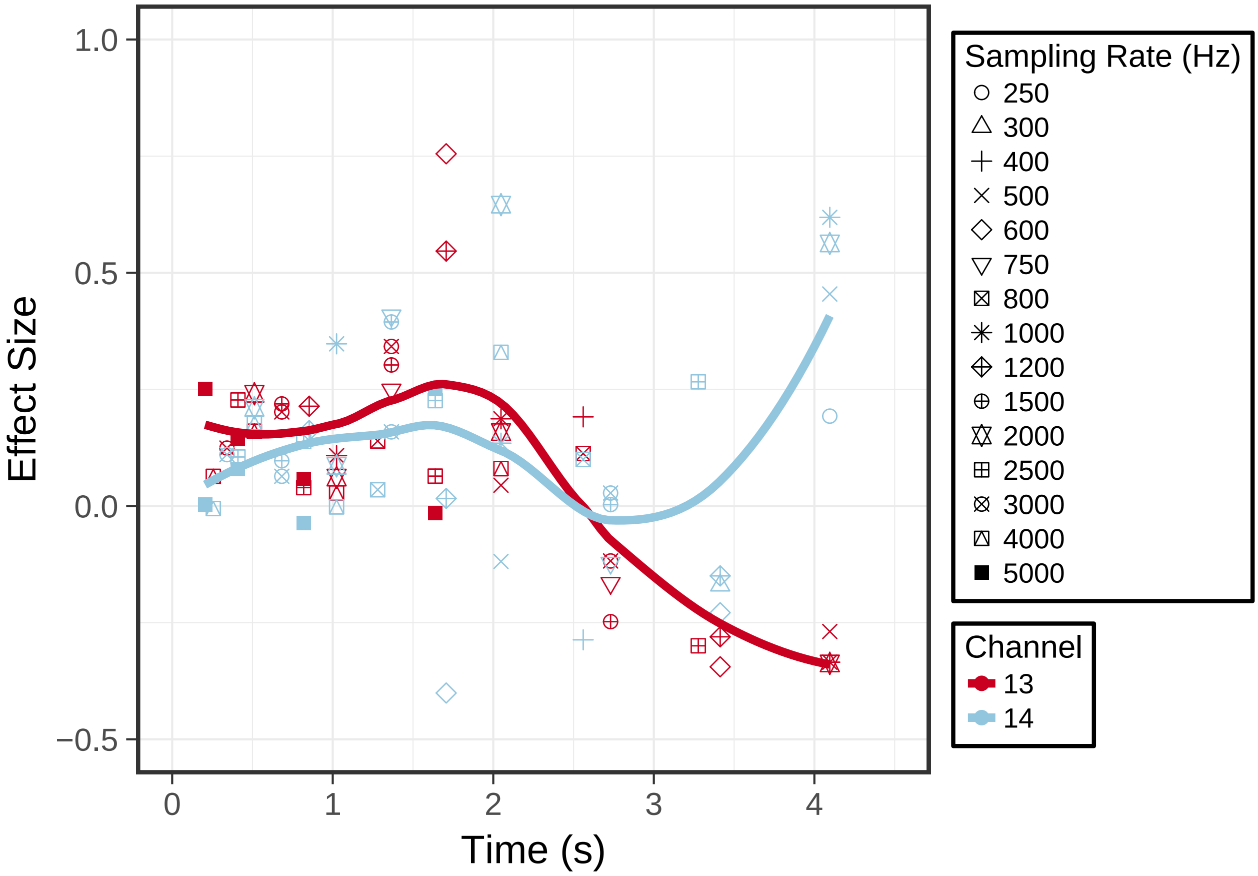

Figure 2: **I001_P034_D01. The two different channels are 'Grid22' and 'Grid23'.**

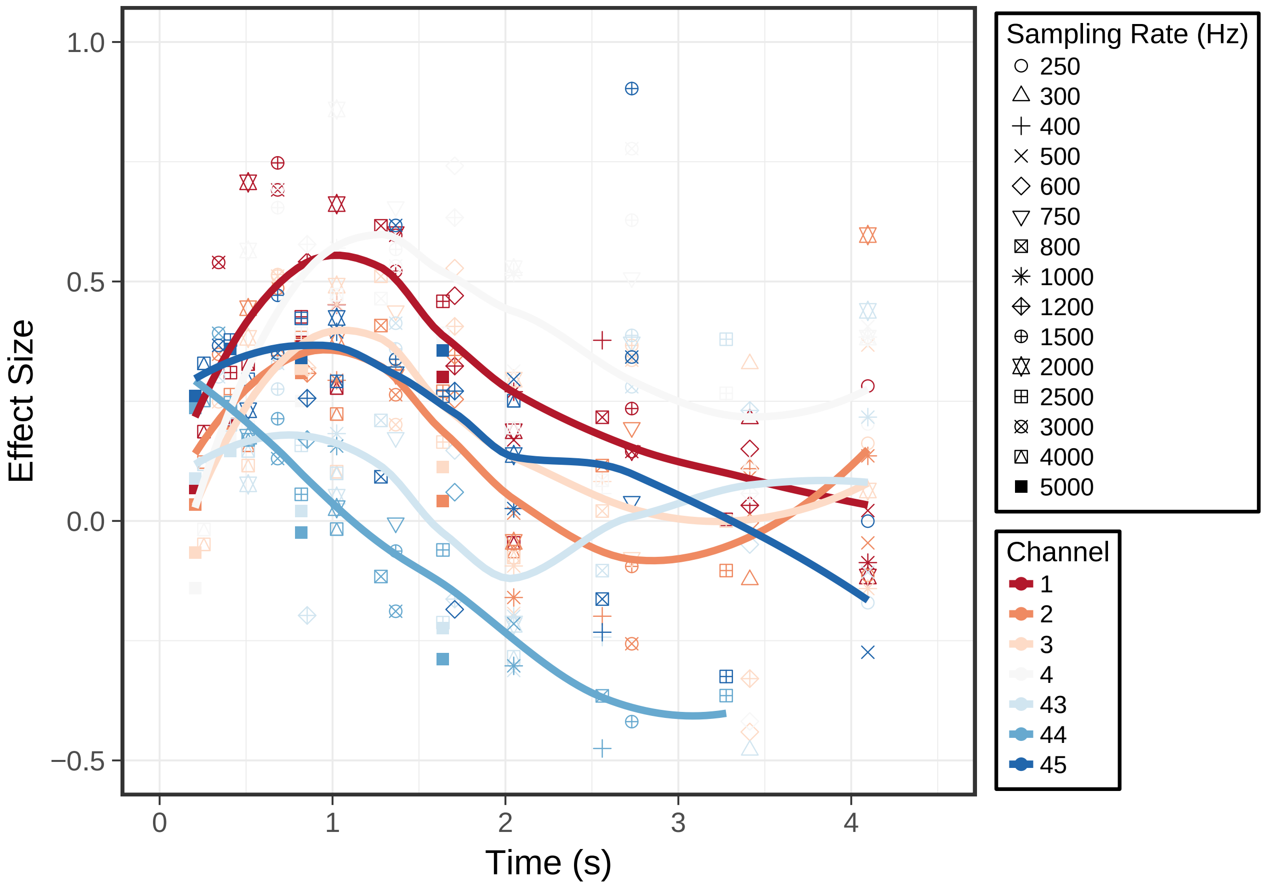

Figure 3: **Study 040. The seven different channels are 'LD1', 'LD2', 'LD3', 'LD4', 'LG44', 'LG45', 'LG46'.**

Appendix C: Multifractal properties of sleep EEG

We applied multifractal analysis to scalp EEG obtained from subjects in a polysomnography set-up. The main goal was to study the impact of the sleep phase on multifractal measures. For this we used the Chhabra-Jensen method on time data that was epoch-wise standardised and sigmoid transformed (i.e. the established pipeline we used in the main manuscript). Each epoch was 10.24 seconds long, and the sampling frequency of the data was 100 Hz giving 1024 points per epoch.

The scalp EEG data studied are from the PhysioNet repository (https://physionet.org/) [51][2] and were described in details by Kemp et al. [54][3]. This article reports analysis performed on signals of five of these patients: 'ST7011J', 'ST7022J', 'ST7041J', 'ST7052J', 'ST7061J'. The signals were recorded at 100 Hz in a polysomnography set-up, this study focused on the available EEG recordings only, with two different signals: Fpz-Cz and Pz-Oz.

Figure 1 shows the variation for $\Delta\alpha^\dagger$ (panel B) and $\Delta f^\dagger$ (panel C) for two EEG channels: Fpz-Cz and Pz-Oz. We also plotted the delta power in Figure 1(D) as reference, which is often a proxy for slow wave sleep stages. To analyse the relationship between the sleep stages (shown in Figure 1(A)) and the multifractal measures, we divided the segments according to the sleep stages. A violin plots shows the distribution of $\Delta\alpha^\dagger$, $\Delta f^\dagger$, and power in the δ band (Fig. 1E-J). A clear drift in the $\Delta\alpha^\dagger$ distribution can be observed that slowly changes from awake to REM to S1, S2, S3 and S4 (Fig. 1E and H). This is in stark contrast to power in the δ band, where the distributions between awake, REM, and S1 are very similar, and then become highly variable in S2, S3 and S4 (Fig. 1G and J).

A dimensionality reduction technique called t-distributed stochastic neighbour embedding (t-SNE) [6] was applied to the six variable collected from the two channels. Fig 2 shows the projection into a two-dimensional space. Note that the information about the sleep stages were not given to the tSNE algorithm. The projection shows one big cluster and two smaller clusters. When applying the information about the sleep stages, it becomes clear that the two smaller clusters are awake states, whereas the one big cluster shows a progression from REM to S4. Note that there are also datapoints from the awake state in this cluster. Future work will show if those datapoints are simply mislabeled, or in any way closer to sleep states. If so, multifractal measures may be useful in devising a classifier for sleep stages in EEG.

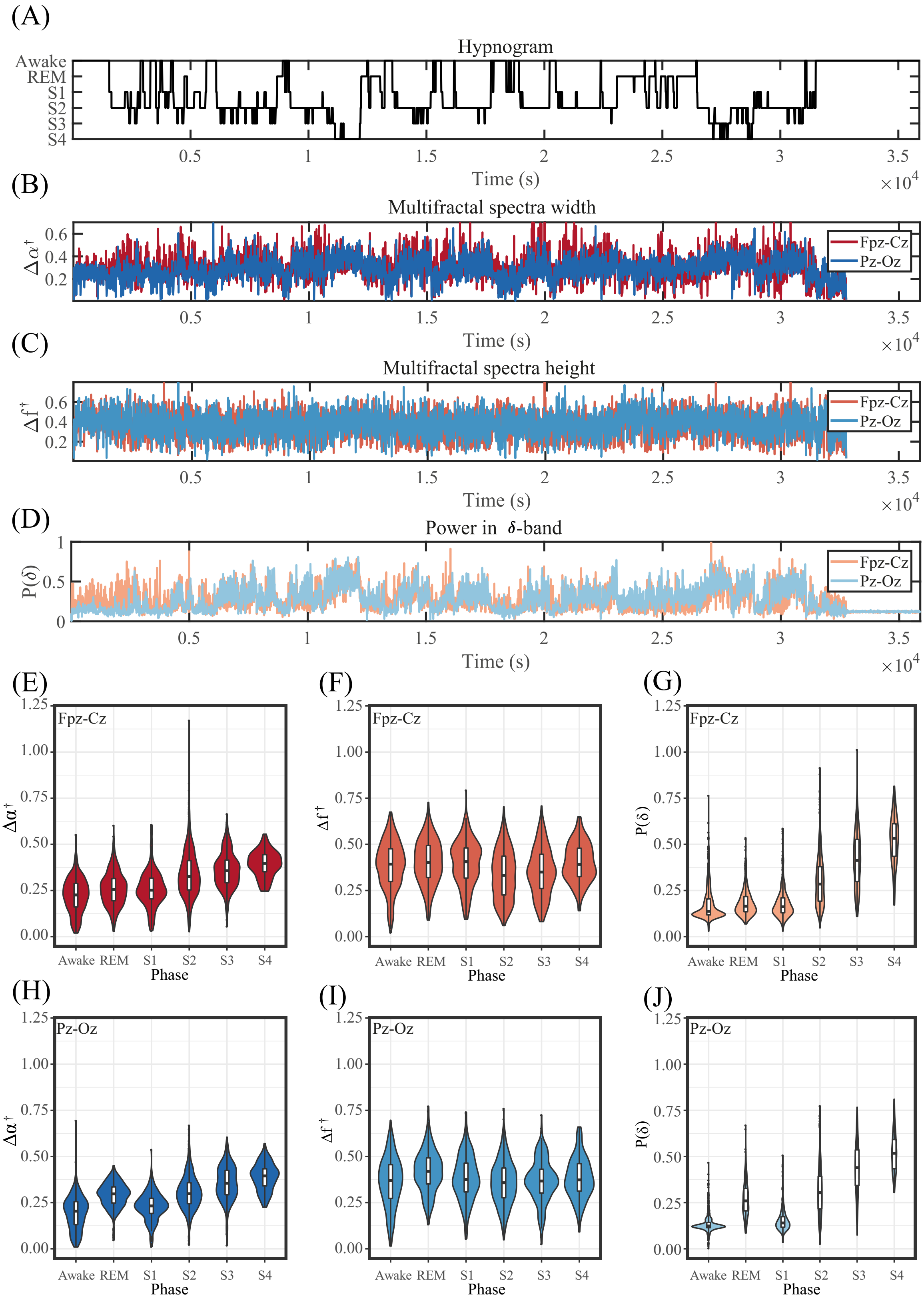

Figure 1: **Impact Sleep phases.** The figure features measures performed on a scalp EEG recorded in a polysomnography in two montages Fpz-Cz and Pz-Oz for patient ST7011J. A) Hypnogram with sleep phases marked by a specialist. B) Variation of the Multifractal spectra width in time. C) Variation of the Multifractal spectra height in time. D) Variation of the power of the δ band in time. E, F, G, H, I, and J) Violin plots of the estimated values of $\Delta\alpha^{\dagger}$ (E, H), $\Delta f^{\dagger}$ (F, I), and $P(\delta)$ (G, J) for both Fpz-Cz (E, F, G) and Pz-Oz (H, I, J) montages. At visual inspection, $\Delta\alpha^{\dagger}$ presents a behaviour similar to $P(\delta)$, following the variations of the hypnogram. The violin plots show an increase in the measures towards more advanced sleep phases for both $\Delta\alpha^{\dagger}$ and $P(\delta)$, in both montages.

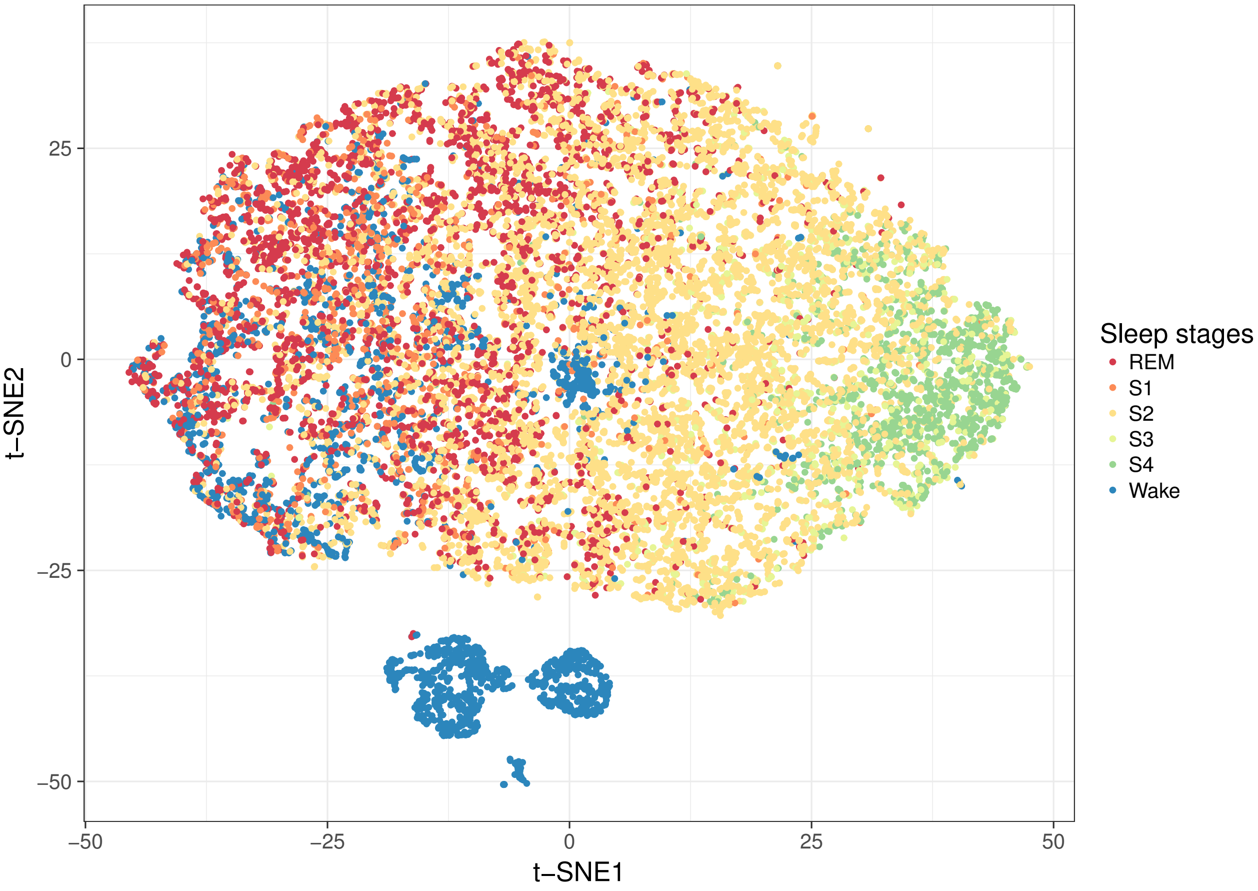

Figure 2: **Dimensionality reduction.** t-distributed stochastic neighbor embedding of the $\Delta\alpha^{\dagger}$, $\Delta f^{\dagger}$, and $P(\delta)$ variables for both Fpz-Cz and Pz-Oz channels in a two dimensional space. The figure shows all sleep stages. It is possible to observe that the measures provide information about the the sleep stage that could be used in order to devise a classifier.

Appendix D: Hausdorff dimension

A formal definition of the Hausdorff dimension is given by Falconer (2003) [1] and it is based on topology and sets theory. Taking U as a generic, non-empty, subset of n-dimensional $\mathbb{R}^n$ space, its diameter can be defined as $|U| = \sup\{|x - y \ : x, y \in U\}$. If $\{U_i\}$ is a countable collection of sets of diameter δ that covers F (a subset of $\mathbb{R}^n$), it is called a δ-cover of F:

$$F \subset \bigcup_{i=1}^{\infty} U_i \quad \text{with} \quad 0 \leq |U_i| \leq \delta \tag{7}$$

Supposing F is a subset of $\mathbb{R}^n$ and S is a non-negative number, for any $\delta > 0$, it is possible to define:

$$\mathcal{H}_\delta^S(F) = \inf\left\{\sum_{i=1}^{\infty} |U_i|^s : \{U_i\} \text{ is a } \delta\text{-cover of } F\right\} \tag{8}$$

$$\mathcal{H}^S(F) = \lim_{\delta \to 0} \mathcal{H}_\delta^S(F) \tag{9}$$

The quantity $\mathcal{H}^S(F)$ is called S-dimensional Hausdorff measure, in which S represents the dimension of the measurement object and will impact on the obtained estimate. For any set $F \subset \mathbb{R}^n$ and $\delta < 1$, it is possible to assert that:

$$\sum_i |U_i|^t \leq \sum_i |U_i|^t \frac{|U_i|^S}{|U_i|^S} \tag{10}$$

$$\sum_i |U_i|^t \leq \sum_i |U_i|^{t-S} |U_i|^S \tag{11}$$

$$\sum_i |U_i|^t \leq \delta^{t-S} \sum_i |U_i|^S \tag{12}$$

And taking the infimum

$$\inf\left\{\sum_i |U_i|^t \leq \delta^{t-S} \sum_i |U_i|^S\right\} \tag{13}$$

$$\mathcal{H}_\delta^t(F) \leq \delta^{t-S} \mathcal{H}_\delta^S(F) \tag{14}$$

Taking the limit when $\delta \to 0$, it is possible to conclude that, if $\mathcal{H}^S(F) < \infty$, $\mathcal{H}^t(F) = 0$ for $t > S$. There is a critical limit for S (S_c) in which $\mathcal{H}^S(F)$ changes from ∞ to 0 as S varies. To this value is given the name of Hausdorff dimension, or Hausdorff-Besicovitch dimension, $S_c = dim_{\mathcal{H}} F$. As shown in figure below.

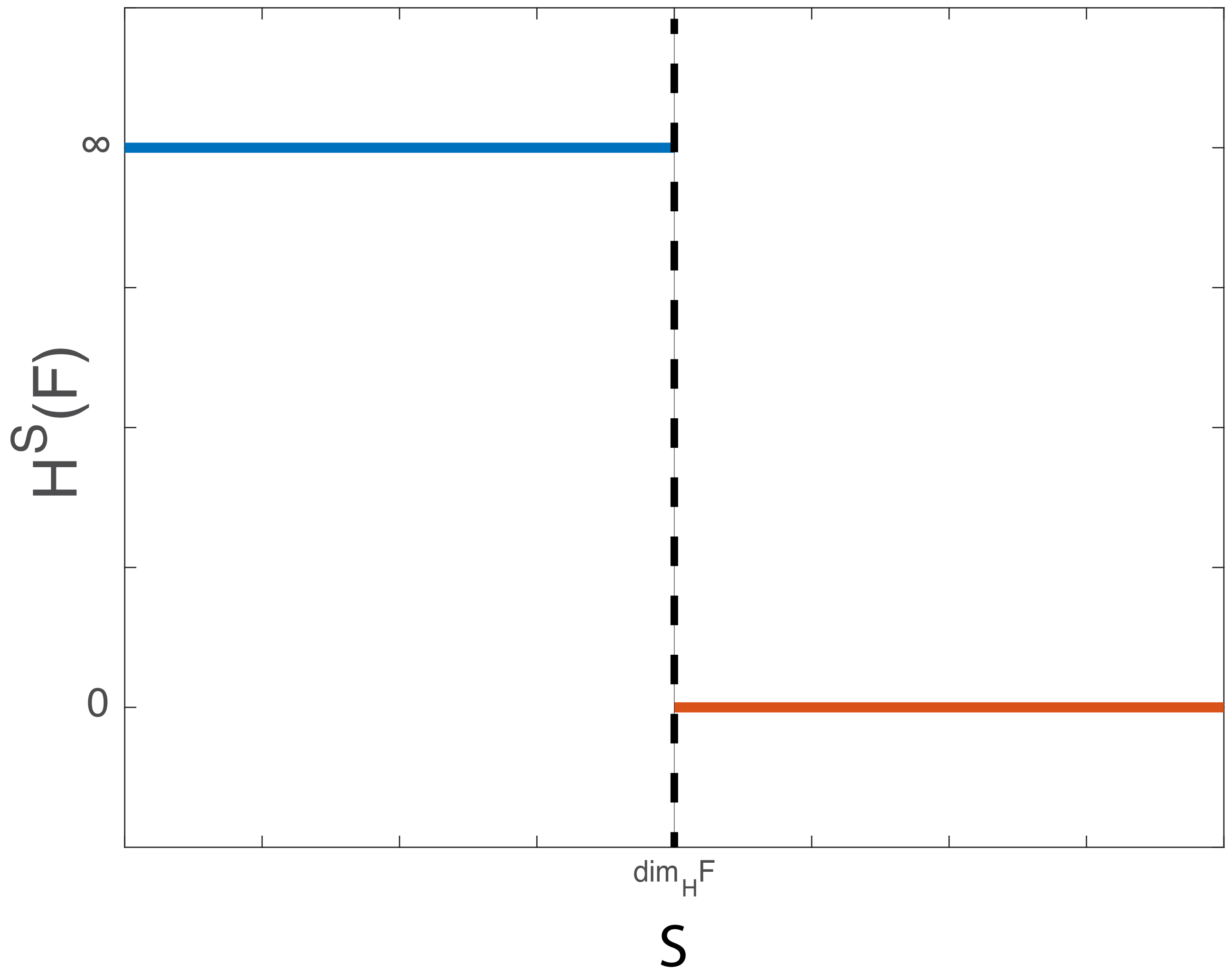

Figure 1: **Transition on the value of the Hausdorff measure $\mathcal{H}^S(F)$ as the measurement object's dimension s changes** . The plot exhibits a transition in which the value of the Hausdorff measure goes from ∞ to 0 for $S_c = dim_{\mathcal{H}}F$

Appendix E

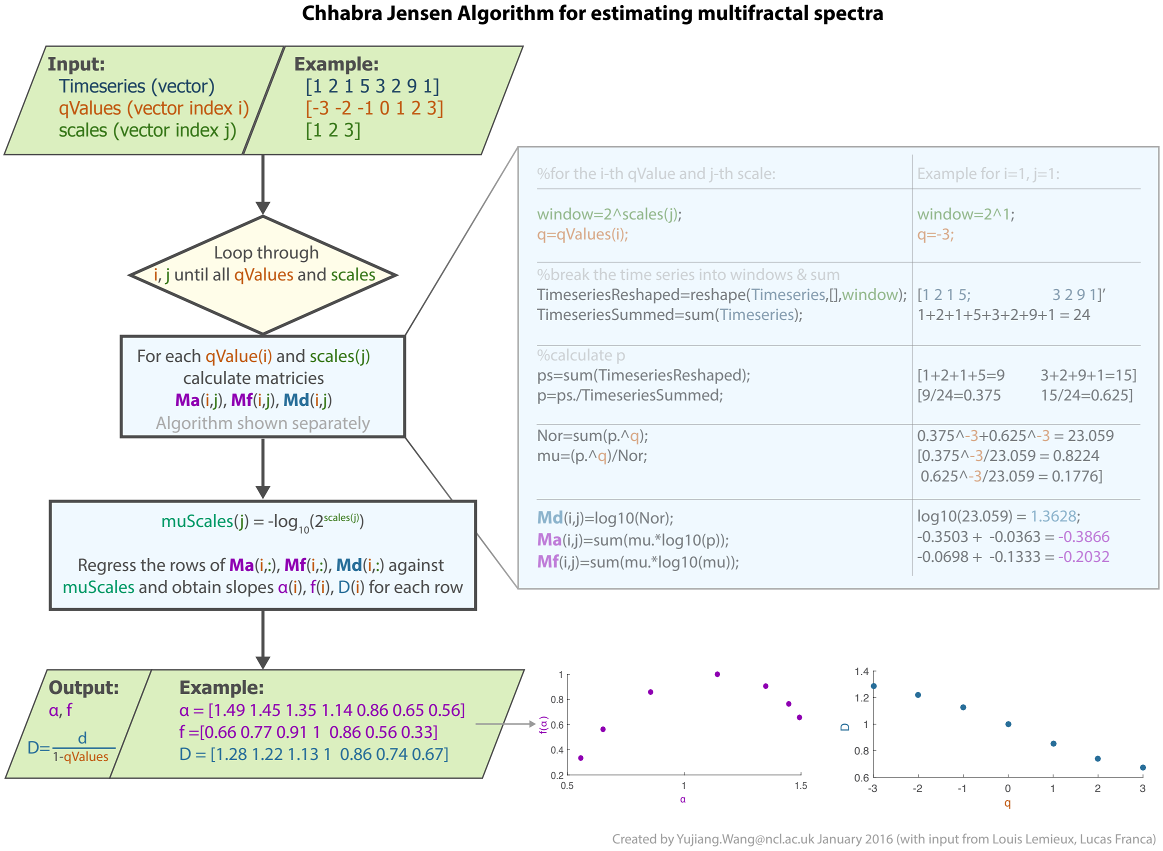

Figure 1: **Flow-chart of the Chhabra-Jensen algorithm.**

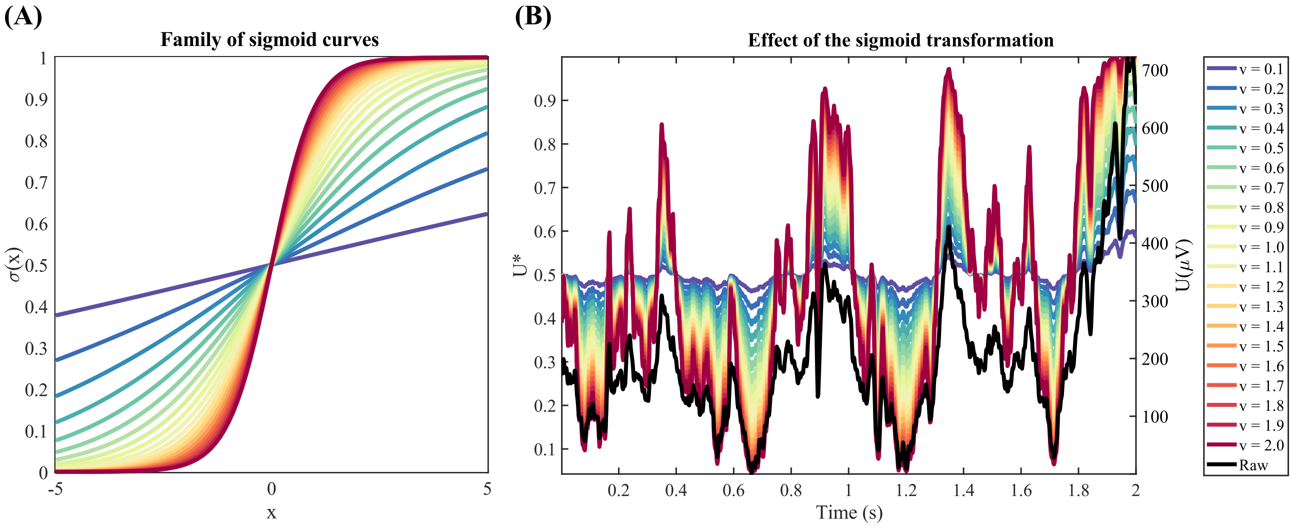

Figure 2: **Impact of sigmoid transformation on the signal.** This function maps the original (raw) time series into a sequence of elements ranging in between 0 and 1, allowing the use of the Chhabra-Jensen method in recordings originally containing negative values. (A) Sigmoid curves with different values of v. This parameter defines how shallow/steep the sigmoid curve will be. (B) Effect of the sigmoid transform on an epoch extracted from an intracranial EEG recording. Smaller values of the parameter v tend to flatten the curve.

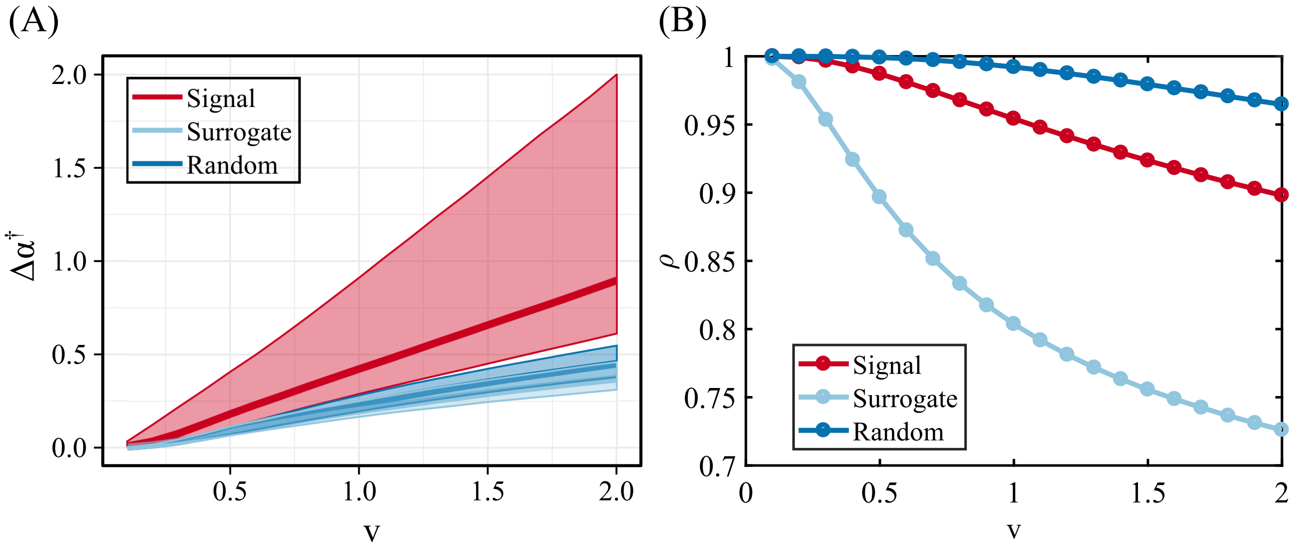

Figure 3: **Assessment of parameters for sigmoid mapping function.** (A) Variability of $\Delta\alpha^{\dagger}$ (0.1, 0.5 and 0.9 quantiles) as a function of the parameter v. (B) Pearson correlation of the original series and the mapped one for the three types of signal (intracranial EEG, its surrogate and a generated random time series). Based on the optimisation criterion - maximum difference between the different signals and minimum distortion (maximum correlation) – the value chosen was $v = 1$.

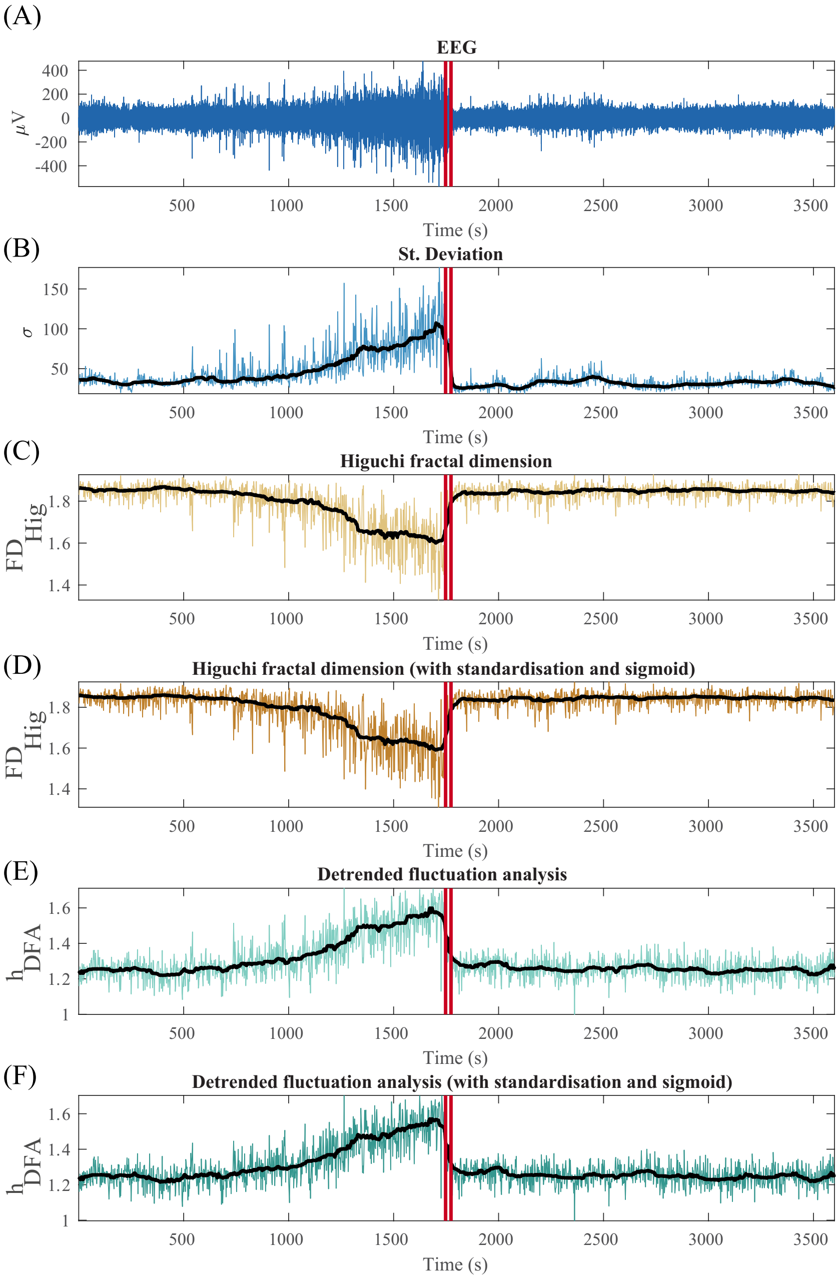

Figure 4: **Monofractal Analysis of seizure segment. Both Higuchi and DFA approaches exhibits similar patterns to the standard deviation of the signal, in accordance with the simulated data presented on the main text of this article. Our signals from intracranial EEG shows a H = 1.2 (non-stationary) for interictal segments in patients with epilepsy. Thus the signal class is fractional Brownian motion (Eke et al., 2000).**

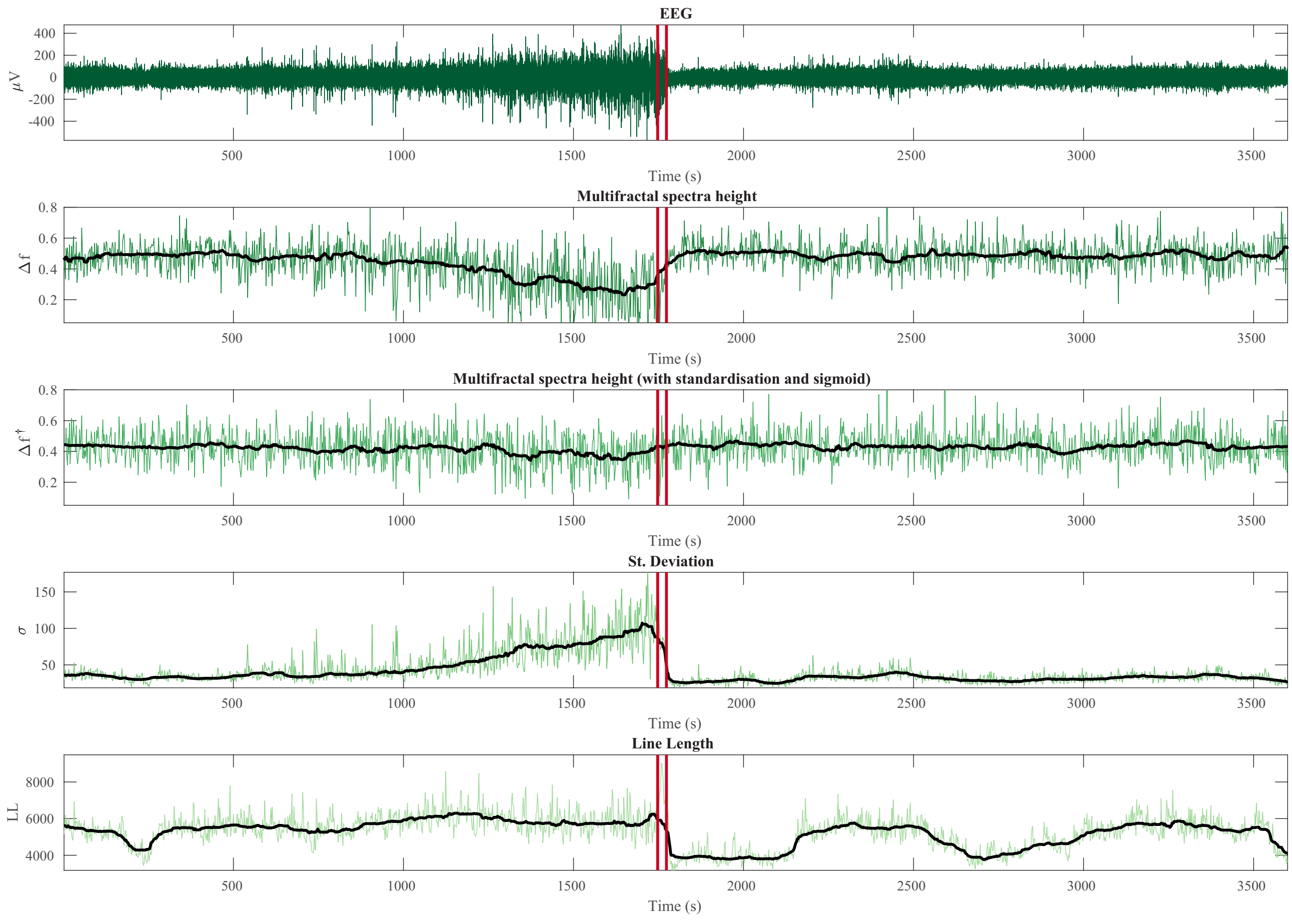

Figure 5: **Multifractal spectrum height and other signal property changes over time.** (A) A single channel intracranial EEG time series segment containing one seizure in the patient NHNN1 (channel 1). The seizure onset and offset are marked by red lines. (B) Variation of multifractal spectrum height ($\Delta\alpha$) estimated on epochs of the EEG segment in (A). (C) Epoch-wise normalised multifractal spectrum height ($\Delta\alpha^{\dagger}$). (D) Standard deviation of the time series for each epoch. (E) Line length of the signal for each epoch.

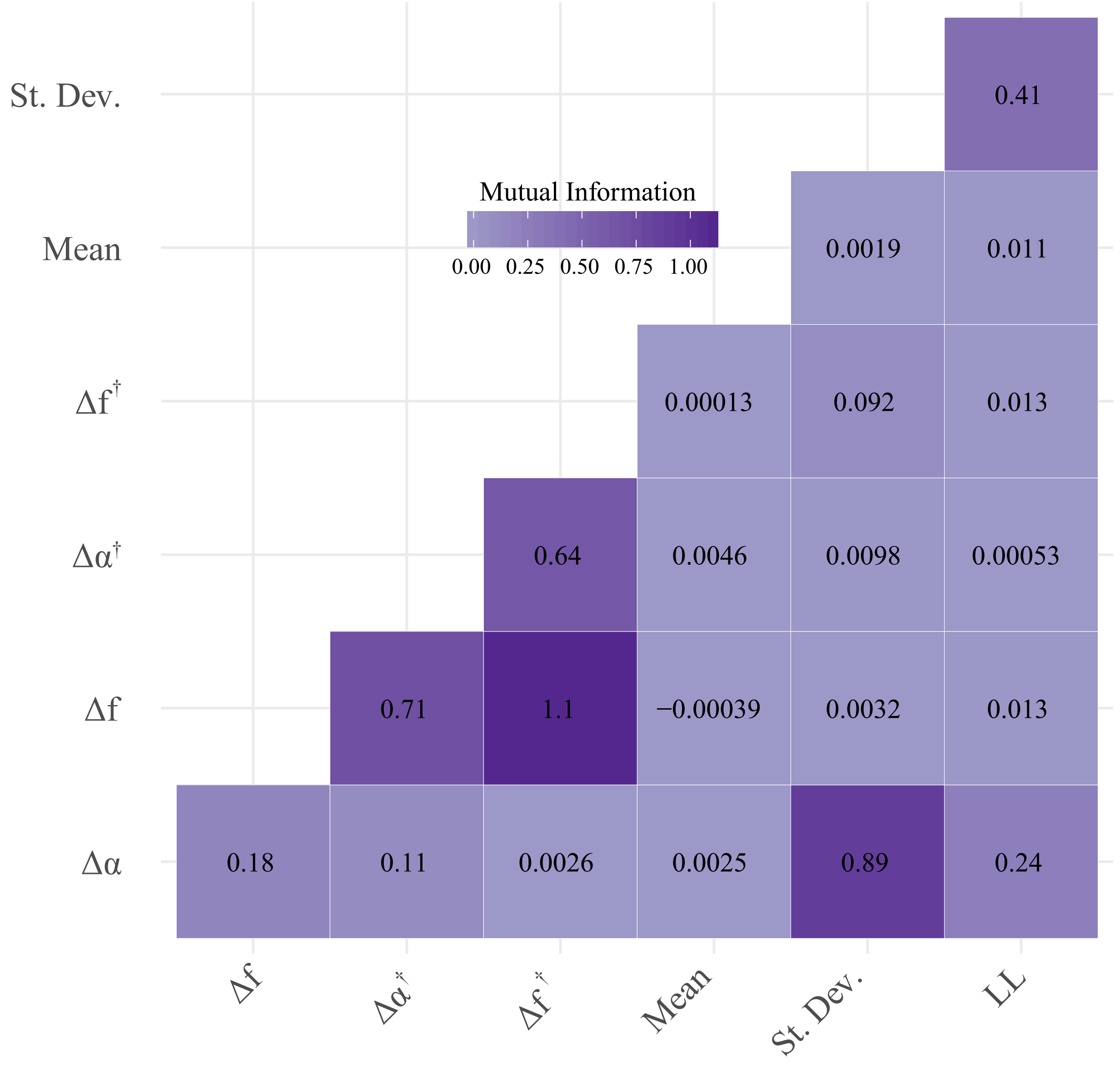

Figure 6: **Mutual Information of different measures.** The MI measure shows higher values for the pair $\Delta\alpha$/St. Dev. than to $\Delta\alpha^{\dagger}$/St. Dev., similar to the result in Fig. 5.

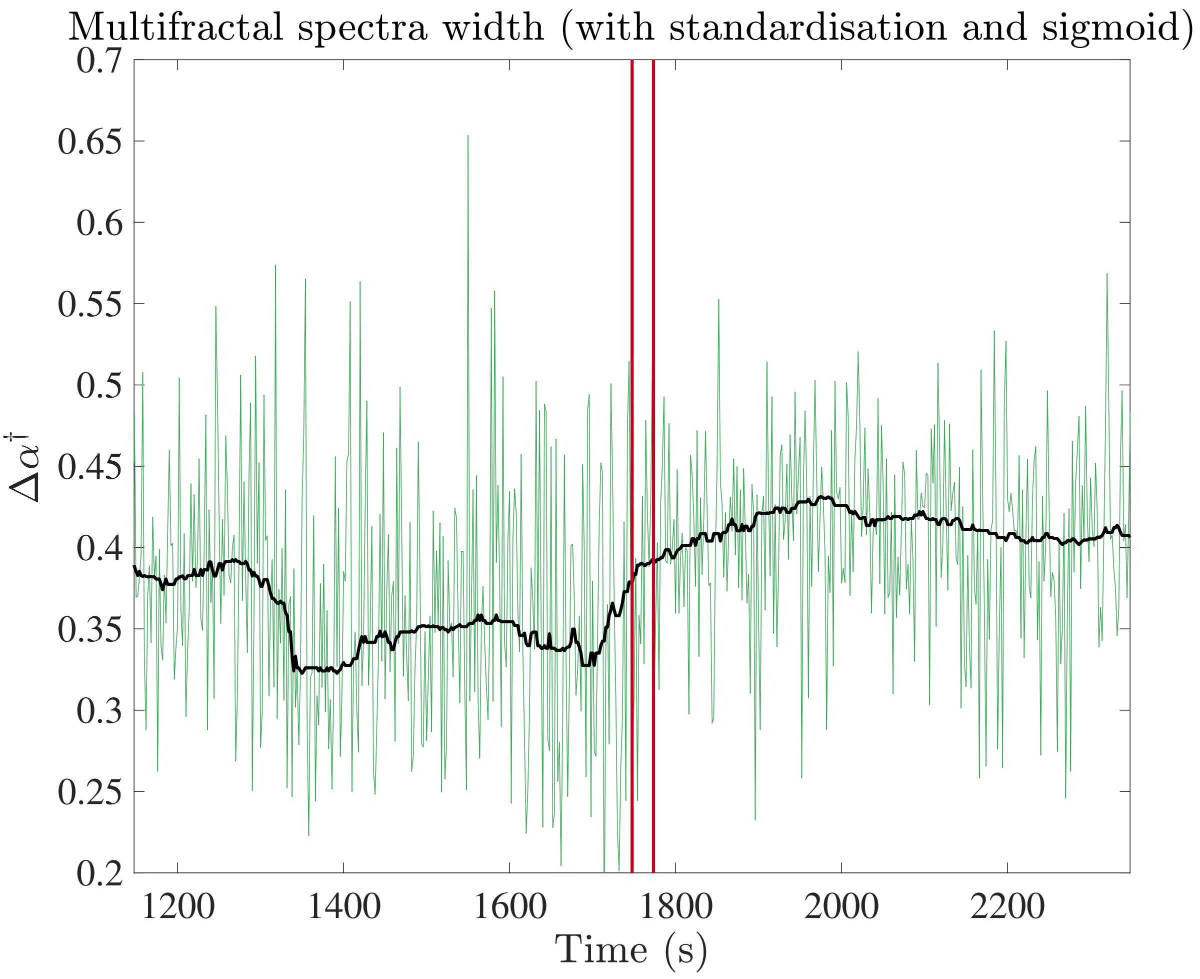

Figure 7: **Multifractal spectrum width ($\Delta\alpha^\dagger$) for a single icEEG channel in the patient NHNN1 (channel 1) around a seizure.** Black line: moving average.

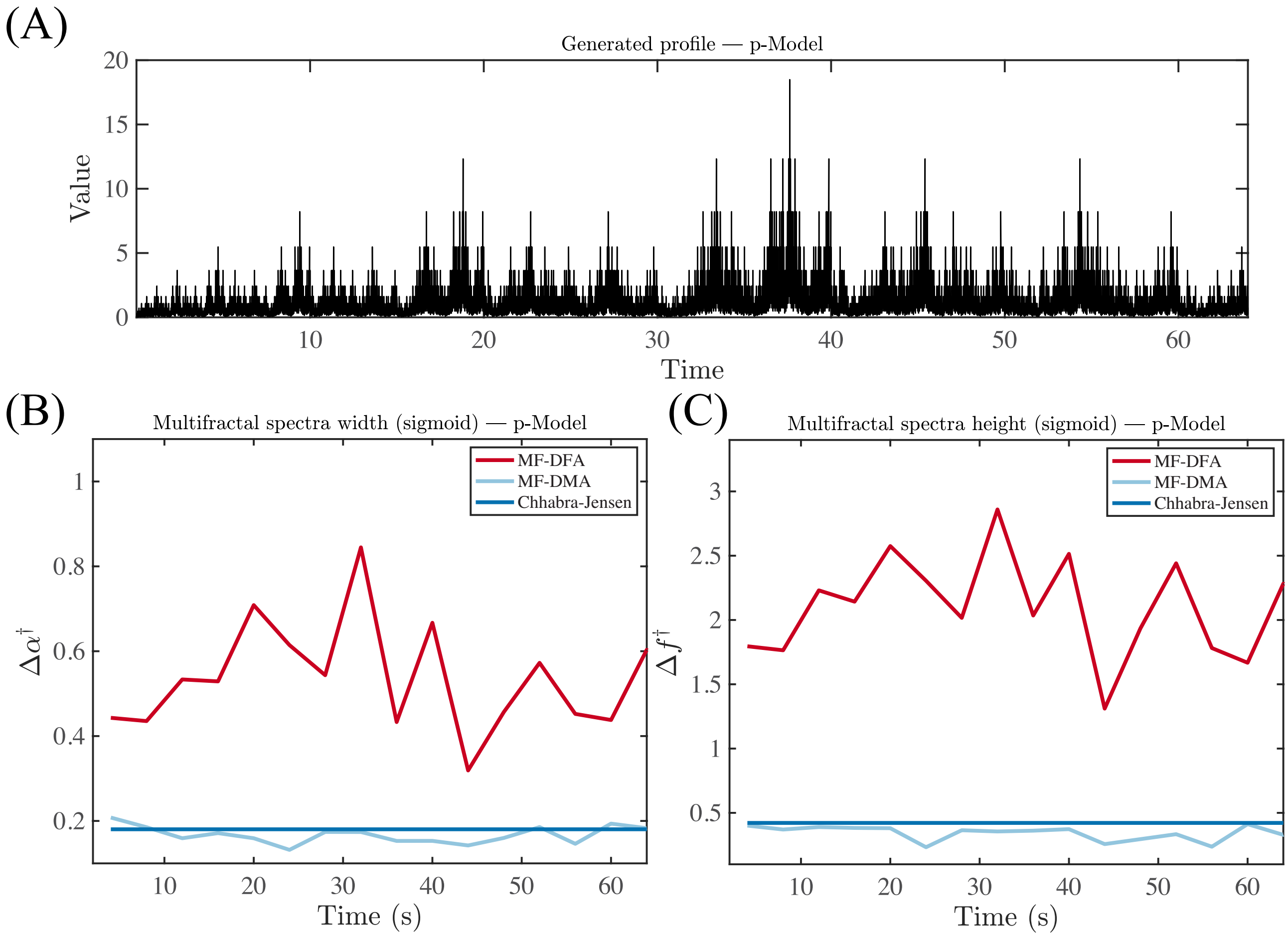

Figure 8: Comparison of three multifractal spectrum estimation methods (with an epoch size of 2048 points) (MF-DFA, MF-DMA and Chhabra-Jensen) for p-Model simulated time series. (A) Time series simulated for $p = 0.4$. (B) Estimated multifractal spectra width $\Delta\alpha^{\dagger}$ and (C) height $\Delta f^{\dagger}$. The result is similar to the one obtained for epochs of 1024 points in Fig. 3.

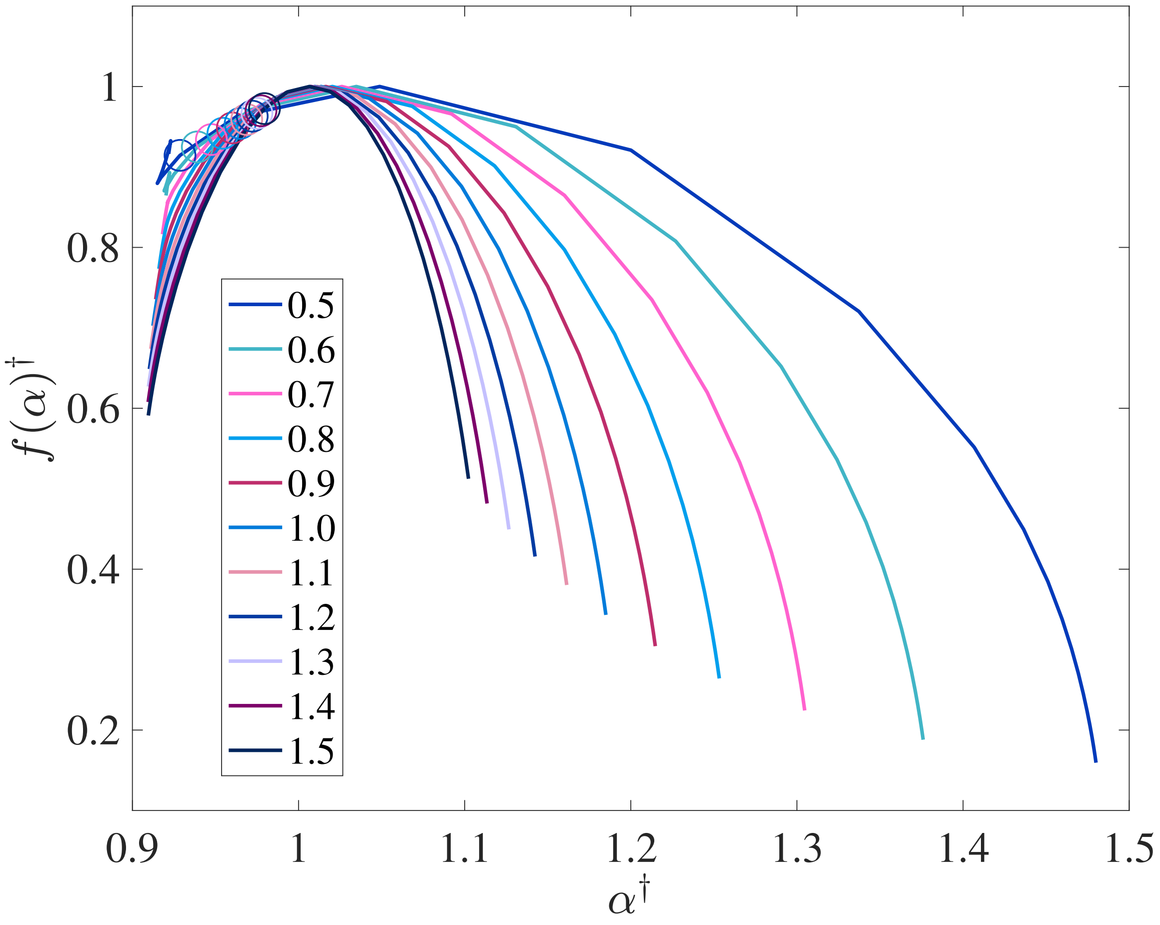

Figure 9: Multifractal spectra from a segment obtained with standardisation procedure, varying the standard deviation s on sigmoid transformation. The standard deviation of the time series (128.4839) was multiplied by a scaling factor ranging from 0.5 to 1.5 by steps of 0.1. The scaling of the standard deviation changes the spectral features however $f(\alpha)$ for $q = 2$ (correlation dimension that is equivalent to monofractal dimension – highlighted by the circles) does not change significantly. The little change in the correlation dimension caused by the standardisation procedure might explain why monofractal methods are not affected by the sigmoid standardisation approach and still correlate with the variance of the signal.

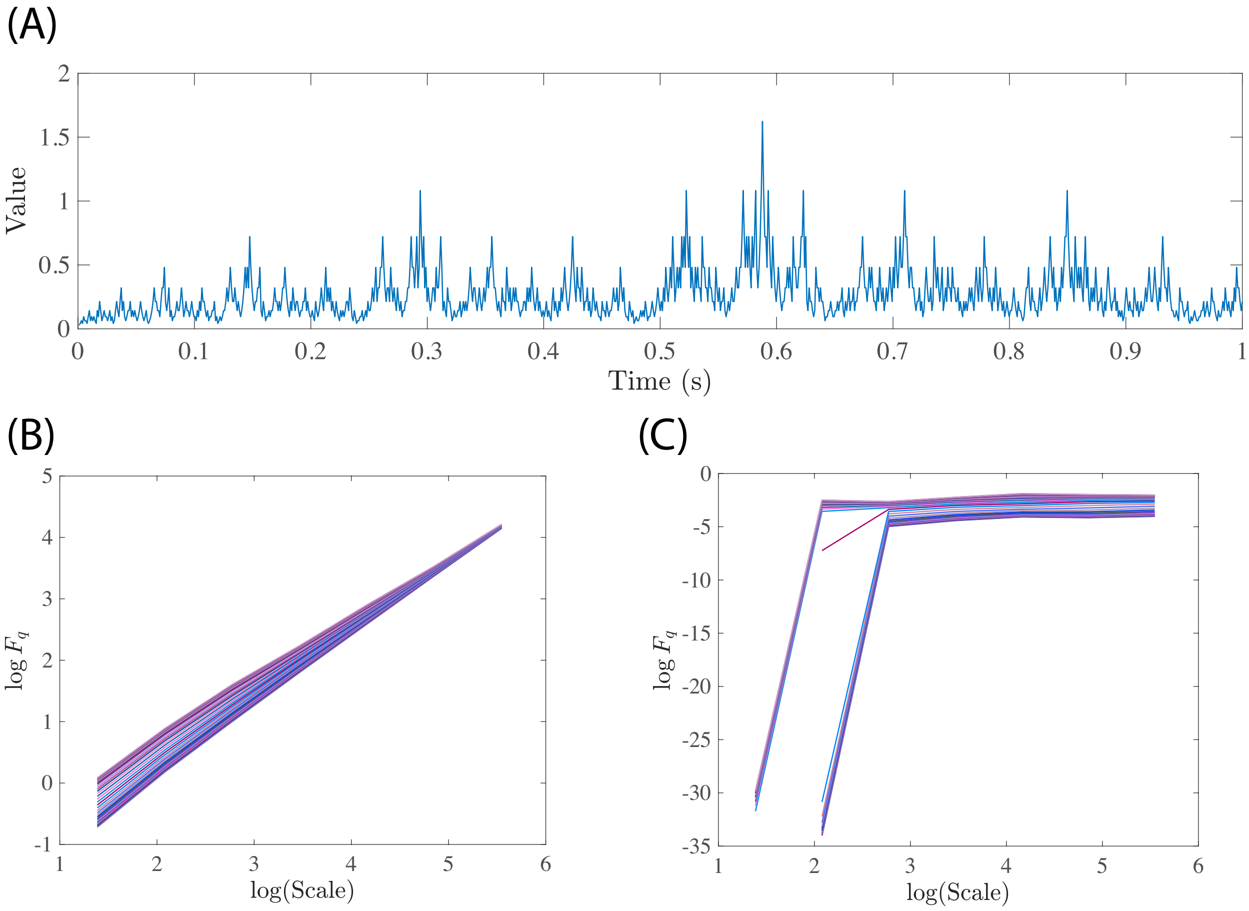

Figure 10: Evaluation of scaling function F for a signal simulated with p-model. The curves were obtained for q ranging between -15 and 15 in unitary steps and scales from 4 to 256 points in a dyadic scale. A) Series generate with p-model (p = 0.4). B) Fluctuation function for MF-DMA method. C) Fluctuation function for MF-DFA method. The values obtained for MF-DFA approach do not exhibit a linear pattern in the small scales. Such lack of linearity causes the problems in obtaining the multifractal spectra by MF-DFA; a finding consistent with those reported by Mukli et al. (2015).

Appendix F: Patient information table

Table 1: Patient information table.

ID	Age	Sex	Sampling rate	Total length
NHNN1	28	M	512Hz	1 h
I001_P010_D01	–	-	5000Hz	4 d 7 h 45 m
I001_P034_D01	33	F	5000Hz	1 d 8 h
Study 040	32	M	5000Hz	2 d 23 h
ST7011J	33	F	100Hz	9.97 h
ST7022J	26	F	100Hz	8.54 h
ST7041J	34	F	100Hz	8.66 h
ST7052J	28	F	100Hz	9.12 h
ST7061J	31	F	100Hz	9.05 h

References

[1] Falconer, K. *Fractal Geometry.* John Wiley & Sons, Ltd, Chichester, UK, sep 2003.

[2] Goldberger, A. L., Amaral, L. A. N., Glass, L., Hausdorff, J. M., Ivanov, P. C., Mark, R. G., Mietus, J. E., Moody, G. B., Peng, C., and Stanley, H. E. PhysioBank, PhysioToolkit, and PhysioNet : Components of a New Research Resource for Complex Physiologic Signals. *Circulation 101*, 23 (jun 2000), e215–e220.

[3] Kemp, B., Zwinderman, A., Tuk, B., Kamphuisen, H., and Oberye, J. Analysis of a sleep-dependent neuronal feedback loop: the slow-wave microcontinuity of the EEG. *IEEE Transactions on Biomedical Engineering 47*, 9 (2000), 1185–1194.

[4] Kroese, D. P., and Botev, Z. I. Spatial Process Simulation. In *Lecture Notes in Mathematics*, V. Schmidt, Ed., vol. 2120 of *Lecture Notes in Mathematics*. Springer International Publishing, Cham, 2015, pp. 369–404.

[5] Shevchenko, G. Fractional brownian motion in a nutshell. Cited 28 July 2017, 2015.

[6] Van Der Maaten, L. J. P., and Hinton, G. E. Visualizing high-dimensional data using t-SNE. *Journal of Machine Learning Research* (2008).
